# Comprehensive Characterisation of the Ketoprofen-β-Cyclodextrin Inclusion Complex Using X-ray Techniques and NMR Spectroscopy

**DOI:** 10.3390/molecules26134089

**Published:** 2021-07-05

**Authors:** Katarzyna Betlejewska-Kielak, Elżbieta Bednarek, Armand Budzianowski, Katarzyna Michalska, Jan K. Maurin

**Affiliations:** 1 Department of Synthetic Drugs, National Medicines Institute, Chełmska 30/34, 00-725 Warsaw, Poland; k.kielak@nil.gov.pl; 2 Falsified Medicines and Medical Devices Department, National Medicines Institute, Chełmska 30/34, 00-725 Warsaw, Poland; e.bednarek@nil.gov.pl (E.B.); j.maurin@nil.gov.pl (J.K.M.); 3 National Centre for Nuclear Research, A. Sołtana 7, 05-400 Otwock, Poland; Armand.Budzianowski@ncbj.gov.pl

**Keywords:** KP/β-CD inclusion complex, NMR, XRPD, crystal and molecular structure

## Abstract

Racemic ketoprofen (KP) and β-cyclodextrin (β-CD) powder samples from co-precipitation (**1**), evaporation (**2**), and heating-under-reflux (**3**) were analysed using X-ray techniques and nuclear magnetic resonance (NMR) spectroscopy. On the basis of NMR studies carried out in an aqueous solution, it was found that in the samples obtained by methods **1** and **2,** there were large excesses of β-CD in relation to KP, 10 and 75 times, respectively, while the sample obtained by method **3** contained equimolar amounts of β-CD and KP. NMR results indicated that KP/β-CD inclusion complexes were formed and the estimated binding constants were approximately 2400 M^−1^, showing that KP is quite strongly associated with β-CD. On the other hand, the X-ray single-crystal technique in the solid state revealed that the (*S*)-KP/β-CD inclusion complex with a stoichiometry of 2:2 was obtained as a result of heating-under-reflux, for which the crystal and molecular structure were examined. Among the methods used for the preparation of the KP/β-CD complex, only method **3** is suitable.

## 1. Introduction

Nonsteroidal anti-inflammatory drugs (NSAIDs) are one of the most commonly used medicine groups due to their analgesic, anti-inflammatory, and antipyretic effects, but NSAIDs also offer protection against diverse critical disorders, including cancer and heart attacks [1]. Ketoprofen (KP) (2*RS*)-2-(3-benzoylphenyl)propionic acid [2,3] is a potent anionic NSAID of the aryl propionic family. The enantiomer of KP responsible for its analgesic effect is dexketoprofen [(2*S*)-2-(3-benzoylphenyl)propionic acid].

KP is an effective NSAID in pain relief and inflammation decrease and is widely used in patients with rheumatoid arthritis and osteoarthritis, gout disorders and other painful conditions [4]. KP is available in both systemic (oral, suppository, or injection) and local topical (patches, compresses, ointments, gels, creams, and lotions) formulations [5]. KP is used in the form of capsules or tablets in doses up to 300 mg/day, and has a short elimination half-life (1.5–4 h); therefore, it is administered orally four times daily. As a result, chronic use may cause serious gastric side effects, such as ulcerations and even gastrointestinal bleeding [6]. Therefore, various approaches have been adopted to improving patient compliance by allowing once-daily oral administration of KP. Such a new sustained-release formulation named Ibifen^®^ has been developed by Roda et al. [5] to gradually release KP within 24 h and ensures therapeutic plasma concentration for the entire period. Recently, an updated evaluation of the multisystemic toxicity of NSAIDs was presented to better understand the side effects of NSAIDs in organ damage [1].

Another limitation is the low water solubility of KP (0.13 mg/mL) [7]. According to the Biopharmaceutics Classification System (BCS) and current guidance, KP is a weak acid assigned to BCS II class [8]. 

Cyclodextrins (CDs) are widely described in the scientific literature [8,9,10,11,12] The most popular among them is β-CD, a cyclic oligosaccharide formed by seven D-glucose units linked by α-(1,4) bonds, resulting in a hollow truncated cone shape. It has a hydrophobic cavity capable of including lipophilic molecules [13]. β-CD has an internal cavity shaped like a truncated cone approximately 8 Å deep and 6.0–6.4 Å in diameter, and presents a narrow rim containing seven primary hydroxyl groups on the C6 atom of glucose and a wide rim with 14 secondary hydroxyl groups positioned on the C2 and C3 carbon atoms. This cavity possesses a relatively low polarity, so it can accommodate organic guest molecules inside [10]. The inclusion of drugs into the CD cavity is mainly driven by hydrophobic interaction, van der Waals forces, hydrogen bonds and the release of water from inside the CD [11].

CDs, by forming an inclusion complex, cause beneficial changes in the characteristics of the guest molecule, such as increased solubility, enhanced dissolution properties, improved stability and reduced side effects [12,14,15]. In this context, in order to increase the solubility of KP, β-CD and hydroxypropyl β-CD (HP-β-CD) were used by shaking method in an aqueous solution at pH 2, 5, 7, and 10 [9]. It was found that the solubility of KP significantly increased with increasing pH and CD concentration; however, the apparent stability constant of the complex was found to decrease with increasing pH due to the decreased affinity of ionised KP to the CD cavity [9]. 

Several methods exist to obtain CD–guest complexes, such as kneading, co-precipitation, freeze-drying, spray-drying, as well as simple grinding in a mortar. The choice of method depends on the properties of the guest and the nature of the chosen CD [16,17]. However, it should be emphasised that the method of preparation of the complex has an influence on the dissolution kinetics, and therefore, it is difficult to make direct comparisons between the systems obtained by other methods.

There are a few crystal structures of inclusion complexes of β-CD with NSAIDs, including diclofenac [18], aspirin [19], ibuprofen [20], flurbiprofen [21], and (*S*)-naproxen [22]; however, only the first two include native β-CD molecules. Naproxen, ibuprofen, and flurbiprofen were studied as complexes with heptakis(2,3,6-tri-O-methyl)-β-CD. Most of the complexes are 1:1 complexes, forming channels in strictly defined directions and with guest molecules occupying the centre of the channels.

KP/β-CD complexation was extensively studied [23,24]. Several studies demonstrated the improved KP solubility and dissolution properties by CD-complexing obtained by grinding, kneading, sealed-heating, and co-lyophilisation of equimolar combinations of KP and CD [7,25]. However, only co-lyophilised and sealed-heating (physical mixtures heated at 80 °C for 3 h) products showed the best dissolution properties. The KP complexes with β-CD and methyl-β-CD were confirmed by differential scanning calorimetry (DSC), Fourier-transform infrared (FTIR) spectroscopy, X-ray powder diffraction (XRPD), and scanning electron microscopy studies [23].

Recently, enantiodiscrimination of KP enantiomers was performed by Obaid et al. [24], who revealed that the (*R*)- and (*S*)-KP enantiomers form inclusion complexes with β-CD in an aqueous solution, and chiral discrimination can be achieved at a neutral pH. For this purpose, spectroscopic techniques (UV, FIR, and Raman) were used. The stoichiometry ratio of 1:1 of the inclusion complexes for both enantiomers were calculated, and values of the binding constants indicated that β-CD form inclusion complexes more preferentially with (*R*)-KP than (*S*)-KP [24]. Currently, there are over 50 different active pharmaceutical ingredients on the market in various types of CD combination formulations, including NSAIDs [26]. The first pharmaceutical product in the form of a prostaglandin E2 complex with β-CD was introduced in Japan in 1976 by the company Ono Pharmaceuticals Co. as Prostarmon E^TM^ sublingual tablets. In the USA, the first approved product was Sporanox^®^ (itraconazole/2-HP-β-CD) in 1997, and in Europe, the first pharmaceutical product in the form of a complex with CD was piroxicam/β-CD, with the trade name Brexin^®^, in 1998 [27], later available under other trade names, such as Cicladon and Flogene. Others include nimesulide/β-CD (Mesulid Fast, Nimedex), diclofenac Na/HP-β-CD (Voltaren Ophtha), ibuprofen/β-cyclodextrin (IBU/β-CD) [28], and indomethacin with both β-CD and HP-β-CD (Indocid) [29]. 

This work is a connecting element in understanding the complete view of the interaction of KP with β-CD, mainly due to the results obtained with the X-ray single-crystal diffraction technique.

The aim of this study was to (i) obtain KP/β-CD inclusion complexes by one or more complexing methods, (ii) grow a single crystal when the XRPD and NMR methods confirmed the formation of the inclusion complexes, and (iii) comprehensively investigate the inclusion complex of KP/β-CD using XRPD, NMR, and X-ray monocrystalline technique. 

In this respect, β-CD is the cheapest, nontoxic type that can be used in a variety of medicinal products for oral, rectal, dermal, and ocular administration, with an acceptable daily intake of 5 mg/kg/day, based on the European Medicines Agency (EMA) document on CDs used as excipients in medicinal products for human use [30]. Although methyl-β-CD yield better performances than β-CD, considering the intrinsic dissolution rate of KP [19], due to its high bioavailability (12%), randomly methylated-β-CD (RM-β-CD) is not approved for oral use. Moreover, RM-β-CD has a longer half-life (t½) compared to other CD derivatives (7h), which is probably related to its ability to interact with cellular membranes, and therefore cannot be used in parenteral products [30]. 

## 2. Materials and Methods

### 2.1. Materials

KP was obtained from the European Directorate for the Quality of Medicines and HealthCare (EDQM, Strasburg, France) and β-CD was purchased from Cyclolab Ltd. (Budapest, Hungary), and both were used without further treatment. Methanol and diethyl ether were purchased from POCH (Gliwice, Poland). Deionised water was obtained from Labconco System Millipore (Bedford, MA, USA). NMR reagents, such as deuterium oxide (D_2_O), isotopic enrichment 99.9% was purchased from ALDRICH (Canada), deuterated 4,4-dimethyl-4-silapentane-1-sulfonic acid (DSS-*d*_6_) was from Wako Chemicals GmbH (Neuss, Germany), and sodium 3-trimethylsilyl tetradeuteropropionate (TSPA-*d*_4_) was from Dr. Glaser AG Basel (Basel, Switzerland). 

### 2.2. Preparation of Complexes

KP/β-CD complexes were formulated by three different methods: co-precipitation **(1)**, evaporation **(2)** and heating-under-reflux **(3)**, as described in the literature for piroxicam/β-CD complexes [31].

#### 2.2.1. Co-Precipitation (1)

KP and β-CD were accurately weighed to result in a 1:1 molar ratio. KP was dissolved in 20 mL of diethyl ether and β-CD was dissolved in 100 mL of water; the two solutions were mixed for 24 h at 28 °C and then cooled to 2 °C. The mixture was filtrated and washed with diethyl ether and dried at 25 °C under vacuum for 24 h.

#### 2.2.2. Evaporation (2)

KP and β-CD (1:1) were dissolved in methanol and stirred for 24 h at 28 °C. The mixture was concentrated under vacuum, filtered, and dried at 25 °C under vacuum for 24 h.

#### 2.2.3. Heating-under-Reflux (3)

KP and β-CD (1:1) were added to 25 mL of distilled water. The mixture was heated under reflux for one hour and then stirred with a magnetic stirrer at room temperature for five days. The solution was concentrated to 10 mL under vacuum and cooled in a refrigerator for one hour, filtered, and dried at 50 °C under vacuum. 

### 2.3. XRPD Studies

**Figure 1:**
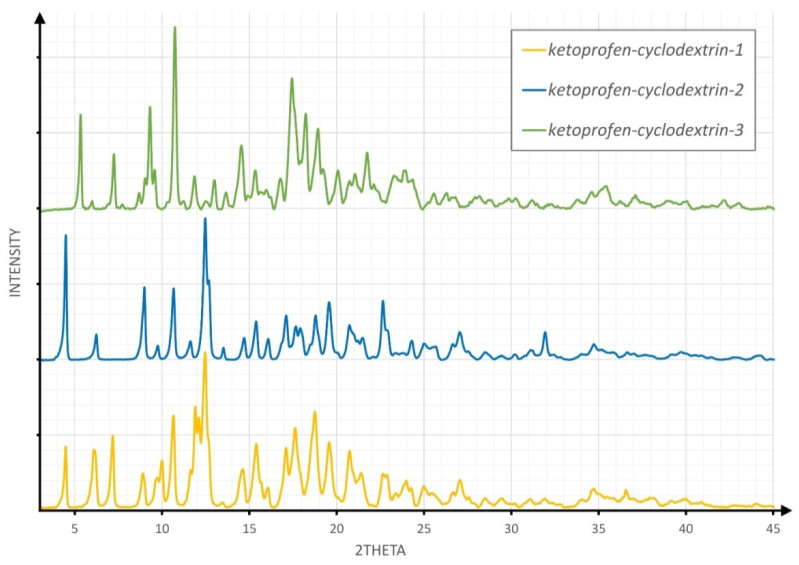
The comparison of X-ray powder diffraction (XRPD) patterns for powder products of three experimental procedures.

Powder samples obtained by three different methods were examined by the XRPD method using a D8 Advance diffractometer from Bruker AXS. The copper type tube was used as the X-ray source in the Bragg–Brentano method using a Våntec linear position-sensitive detector [32]. Data for samples were measured in 2θ range 3–45° with a step of 0.004° and 110s per step scans (Figure 1). This technique is very sensitive to powder crystalline forms. New reflections can indicate a modification of the crystalline phase or some new phases. However, many chemical compounds can exist as different polymorphs, resulting in different powder diffraction patterns for the same chemical composition [33].

#### 2.3.1. Obtaining of Single Crystals

The object of the crystallisation trials was a powder sample (heating under reflux) for which signs of complex formation were observed. Crystallisation was performed by the evaporation method. Several different batches of various concentrations were prepared for this purpose using water and MeOH as solvents. Powder materials were dissolved in solvent mixtures: 30%, 50%, 80%, and 100% MeOH. The single crystal was obtained from 80% MeOH. Crystals were grown for three months at room temperature by slow evaporation. The quality of the obtained crystals was examined under the microscope using polarising light. 

#### 2.3.2. X-ray Single-Crystal Technique

A single crystal with dimensions 0.177 mm × 0.222 mm × 0.451 mm was selected for the X-ray diffraction studies. The experiments were carried out using an Xcalibur R Oxford Diffraction four-circle diffractometer equipped with a copper X-ray tube and a CCD detector. The recorded images were analysed and processed using the CrysAlis software [34] to obtain unit cell dimensions, and corrected intensities of collected reflections. The SIR 2014 programme (Semi-Invariants Representation) [35] was used for structure solution. An Ab-initio crystal structure determination of macromolecules, using a new Vive la Différence (VLD) cyclic phasing algorithm, neither using direct nor Patterson methods [36], was applied. The VLD phasing approach allowed solving of this crystal structure. The VLD procedure was applied to 20 trials performing 300 cycles of electron density modification (EDM) on the Difference Fourier Map in each trial. The X-Seed [37] and ShelXle [38] programmes were used for structure visualisation during the refinement process to rename and sort the order of atoms. The refinement using full-matrix least-squares on F^2^ was performed applying SHELXL-2014/7 and SHELXL-2018/3 software [39].

Due to the lack of data for refinement of all parameters for such a large organic structure at once, several additional procedures in the refinement process were implemented (details are presented in the Supplementary Materials). In the final stages of structure refinement, all positional and anisotropic displacement parameters for all non-H atoms were applied; whereas, for the H atoms, which positions were located using standard geometrical criteria, isotropic temperature factors tied with equivalent isotropic displacement parameters of heavy atoms to which they are bonded to were used. The unit cell dimensions of this crystal and the final refinement parameters are presented in Table 1. Some figures in this work were prepared as autostereograms [40] of the crystal structure to allow the visualisation of flat images with a three-dimensional perspective. The X-Seed and POV-Ray software [37,41] have been used for this purpose. 

### 2.4. NMR Studies 

#### 2.4.1. Instrumentation

The NMR spectra of samples obtained by the three methods were recorded at 298 K in D_2_O solutions by using a Varian VNMRS-500 spectrometer (Varian, Inc., NMR Systems, Palo Alto, CA, USA) operated at 499.8 and 125.7 MHz for ^1^H and ^13^C measurements, respectively. All experiments were run by using the standard Varian software (VnmrJ version 3.1A software, Varian, Inc., NMR Systems, Palo Alto, CA, USA). The spectrometer was equipped with an inverse ^1^H{^31^P-^15^N} 5 mm Z-SPEC Nalorac IDG500-5HT probe (Nalorac Corp., Martinez, CA, USA) with an actively shielded *z*-gradient coil to give a maximum gradient strength of 61.1 G·cm^–1^. The high power ^1^H and ^13^C π/2 pulses were 7.6 and 11.6 μs, respectively.

The ^1^H NMR spectra and the ^1^H dimension in two-dimensional (2D) heteronuclear spectra were referenced to the solvent (D_2_O, *δ*_H_ = 4.76 ppm), which was treated as a secondary standard. The ^13^C dimension in 2D heteronuclear spectra was indirectly referenced [42].

#### 2.4.2. Methodology

NMR standards (e.g., sodium 3-trimethylsilyltetradeuteriopropionate (TSPA-d_4_) or 4,4-dimethyl-4-silapentane-1-sulfonic acid (DSS)) can form inclusion complexes with CDs [43]. Therefore, the concentrations of KP or β-CD in the solutions were determined against quantitatively added DSS, but only after all measurements related to the study of the complexation effects were performed. 

A standard single-pulse experiment was used to acquire the ^1^H NMR spectra in a quantitative regime using an 8000 Hz spectral window, 30° pulse width, an acquisition time of 4.0 s, 256–2048 scans (depending on the concentration), relaxation delay of 15 s, and 64,000 complex data points. For experiment with water suppression, 90° pulse width and a relaxation delay of 2 s were used.

^1^H^−1^H COSY spectra were run by using a spectral width of 6000 Hz in both dimensions, 1024 complex points in t_2_, 512 complex points in t_1_, 1–4 scans per increment, and a relaxation delay of 1 s. 

For ^1^H-^13^C HSQC, we used spectral widths of 6000 Hz in F2 and 19,000 Hz in F1, 1024 complex points in t_2_, 512 complex points in t_1_, four scans per increment, a relaxation delay of 1s and ^1^*J*(C,H) = 146 Hz. 

^1^H-^13^C HMBC was performed using spectral widths 6000 Hz in F2 and 28,500 Hz in F1, 1024 complex points in t_2_, 256 complex points in t_1_, 32 or 128 scans per increment, a relaxation delay of 1s and *^n^J*_(C,H)_ = 8 Hz. 

Pulsed field gradient spin echo (PFGSE) experiments were performed by using the Oneshot (stimulated echo sequence incorporating bipolar gradients) [44,45] pulse sequence with pre-saturation of the residual water signal with 64–2048 transients, 16 dummy scans and a 20% imbalance factor. The diffusion time (*Δ*) and the total diffusion-encoding gradient duration (*δ*) were chosen according to the value of *D* (*Δ* = 80 ms for KP and 100 ms for complexes; *δ* = 2 ms); whereas 16–20 values of the diffusion-encoding gradient were used, incremented from 6.0–50.0 G·cm^−1^ in steps such that the strength of the next gradient was equal to the previous gradient squared. Other parameters included the following: a sweep width of 6000 Hz, 32 k data points, an acquisition time of 2.7 s and relaxation delay of 2 s. The data were processed by use of Varian VnmrJ software, with the option of correction for spatially non-uniform pulsed field gradients.

#### 2.4.3. Calculating Binding Constants from the Diffusion Coefficients

Assuming that the binding equilibrium in the NMR experiment is established very quickly (dynamic equilibrium method), and complexes exist with a stoichiometry of 1:1 (1), the binding behaviour can be described with the following mathematical model:

CD + L ↔ CD·L

(1) $$K_{a}=\frac{\left[\mathrm{CD}\cdot L\right]}{\left[\mathrm{CD}\right]\left[L\right]}\;(1:1\;\mathrm{complex})$$

in which CD is β-CD; L is KP; [CD·L], [CD], and [L] are the equilibrium concentrations of complex 1:1, CD and L, respectively; and *K_a_* is the binding constant.

Binding constants (*K_a_*) of the complexes were estimated by analysis of the diffusion coefficient of β-CD (CD) and KP (L) as a function of the host and guest concentration [46], according to Equation (1). The *K_a_* may be extracted from diffusion experiments as follows. In the case where the exchange rate between the uncomplexed and complexed species is fast on the NMR timescale, the observed diffusion coefficients (D, [m^2^ s^–1^]) are a weighted average of the diffusion coefficients of the uncomplexed and complexed forms, in which the weighting factors are the relative population sizes of the respective forms. Thus, the observed diffusion coefficients may be expressed as:

*D*_OBS-L_ = *MF*_L_*D*_L_ + (1 − *MF*_L_)*D*_[CD·L]_ (2)

*D*_OBS-CD_ = *MF*_CD_*D*_CD_ + (1 − *MF*_CD_)*D*_[CD·L]_ (3)

in which *D*_OBS-L_ and *D*_OBS-CD_ are the observed averaged diffusion coefficients for L and CD; *D*_L_ and *D*_CD_ are the diffusion coefficients for uncomplexed L and uncomplexed CD; *MF*_L_ and *MF*_CD_ are the molar fractions of uncomplexed L and uncomplexed CD in the solution containing both molecules; and *D*_[CD·L]_ is the diffusion coefficient for the complex.

Equation (1) can be also expressed as:

*K*_a_ = [CD·L]/(*C*_CD_ − [CD·L])(*C*_L_ − [CD·L]) (4)

in which *C*_CD_ and *C*_L_ are the initial concentrations of CD and L. The unknown complex concentration can be calculated from equations:

[CD·L] = (1 − *MF*_CD_)*C*_CD_ (5)

[CD·L] = (1 − *MF*_L_)*C*_L_ (6)

In the case where the host molecule is much larger than the guest, it can be assumed that the diffusion coefficient of the host–guest complex is the same as that of the host molecule (D_[CD·L]_ ≅ D_OBS-CD_). However, this formal treatment of the data includes a simplification that may cause that the results are affected by an error. 

## 3. Results and Discussion 

### 3.1. XRPD Studies

Comparison of the XRPD patterns of separate KP and β-CD powders with the resulted solid obtained in the given experimental procedure (**1–3**) enabled verification of the inclusion process. In general, it was expected that the observation of new reflections was the result of new phase formation, while the diffraction pattern, consisting of a superposition of characteristic reflections of both substrates, led to the conclusion that a simple physical mixture of substrates was formed.

Observation of a series of new reflections that were not observed for either KP or β-CD served as positive evidence of inclusion. The comparison of the XRPD results for powder samples obtained by the three different methods is shown in Figure 1. 

Each type of experiment described yielded a powder that resulted in a different diffraction pattern and therefore, a different product.

**Figure 2:**
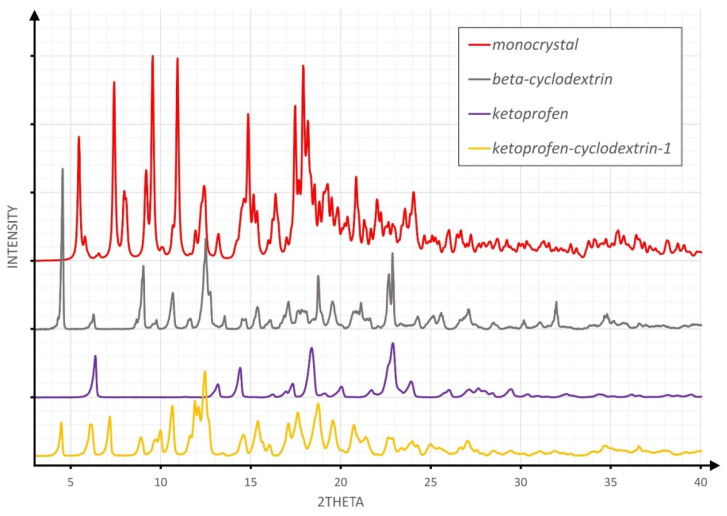
The comparison of XRPD patterns for the product of the co-precipitation experiment.

**Figure 3:**
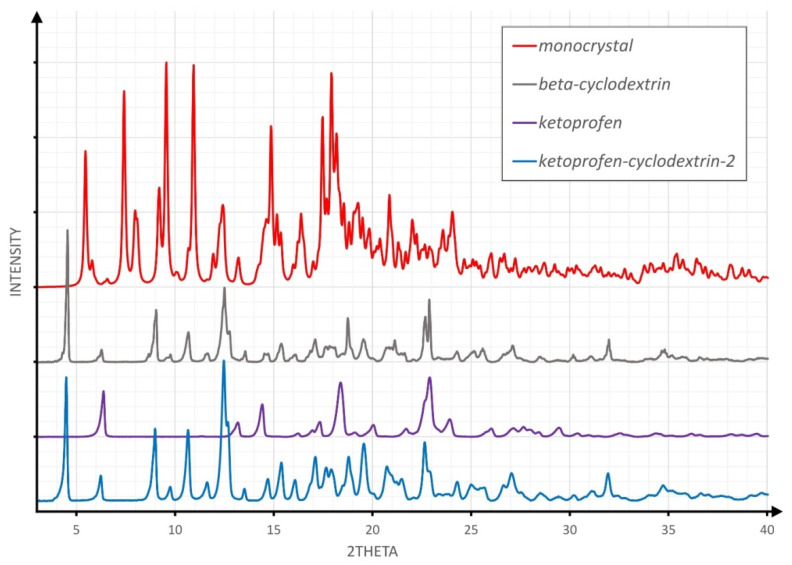
The comparison of XRPD patterns for the product of the evaporation experiment.

**Figure 4:**
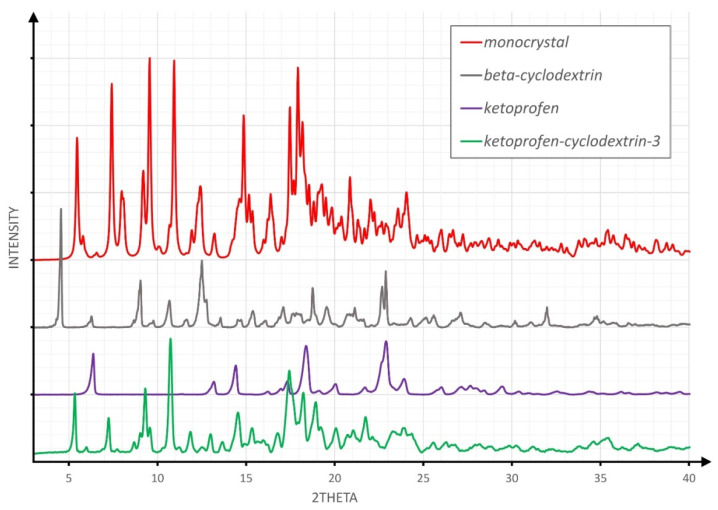
The comparison of XRPD patterns for the product of the heating-under-reflux experiment.

Figure 2, Figure 3 and Figure 4 overlay the diffraction patterns of KP, β-CD, and the corresponding product designated as KP-CD-X, in which X is 1–3, respectively, corresponding to the given method of experimental preparation of the complexes. Each diffraction pattern was subjected to the standard procedure of smoothing and background subtraction. Additionally, the upper part of the figures shows the powder diffraction pattern calculated for the single-crystal structure of the complex.

The inspection of Figure 2, Figure 3 and Figure 4 led to the conclusion that the diffraction patterns shown on the bottom of Figure 2 and Figure 4 differ substantially from the diffraction patterns of both KP and β-CD, which might suggest an inclusion complex formation. However, both powders are different from each other, as shown in Figure 1. The diffraction pattern shown in Figure 3 (pale blue drawing) probably represents a physical mixture of both components, as all diffraction maxima shown in the blue pattern are observed in either the grey (β-CD) or violet (KP) diffractogram.

Another conclusion that might be drawn from Figure 4 is that heating-under-reflux results in a product similar to the complex observed in a monocrystal, as the angular positions of the observed reflections in the green pattern are very close to those of the red pattern.

The most complicated case represents the powder from co-precipitation, presented in Figure 2. Although new reflections are present (corresponding neither to β-CD nor KP), some reflection positions are close to those of single components. This might be the result of the formation of a different inclusion complex, as well as of a mixture of the former complex and not transformed substrates. Unfortunately, in the present stage of the research, such uncertainty has not been resolved.

### 3.2. X-ray Single-Crystal Analysis

The compound crystallised in the monoclinic crystal system in *P*2_1_ space group with two molecules (Z = 4) in the unit cell. In the independent part of the unit cell, there are two independent molecules of β-CD and two molecules of KP. The two β-CD molecules are connected to each other by a series of hydrogen bonds between hydroxyl groups (Figure S1 in Supplementary Material). In this respect, the presented structure is slightly similar to the aspirin/β-CD complex [19], which is composed of two molecules of aspirin and two molecules of β-CD. The structure also contains an additional flat salicylic acid molecule in the gap between two β-CD macrocycles.

**Figure 5:**
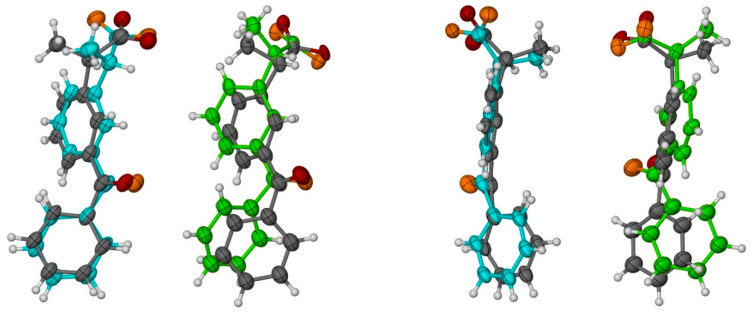
Two views of ketoprofen (KP) conformations of molecules A (blue) and B (green) observed in the crystal structure. The less occupied conformations (C,D, respectively) are shown in grey. Oxygen atoms in molecules A and B are shown in orange. All non-H atoms are shown as 50% probability ellipsoids.

**Figure 6:**
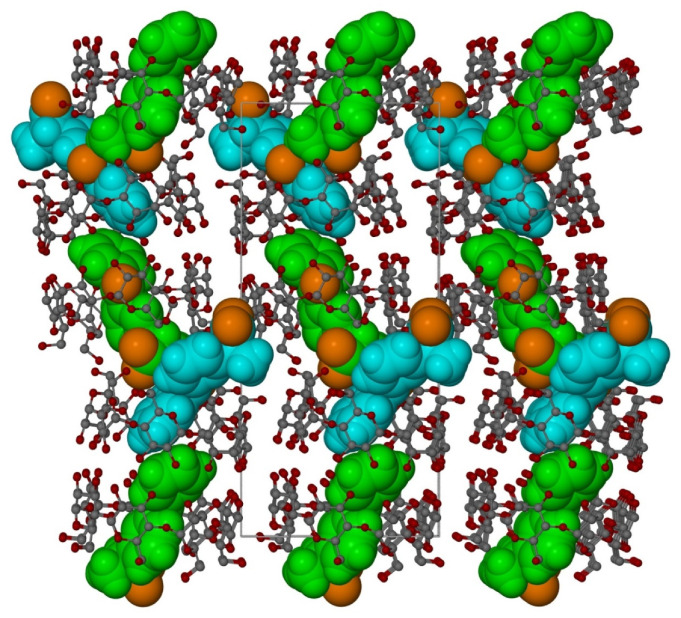
Autostereogram of the crystal structure in c-direction. Only one KP conformation for every blue and green molecule is shown.

**Figure 7:**
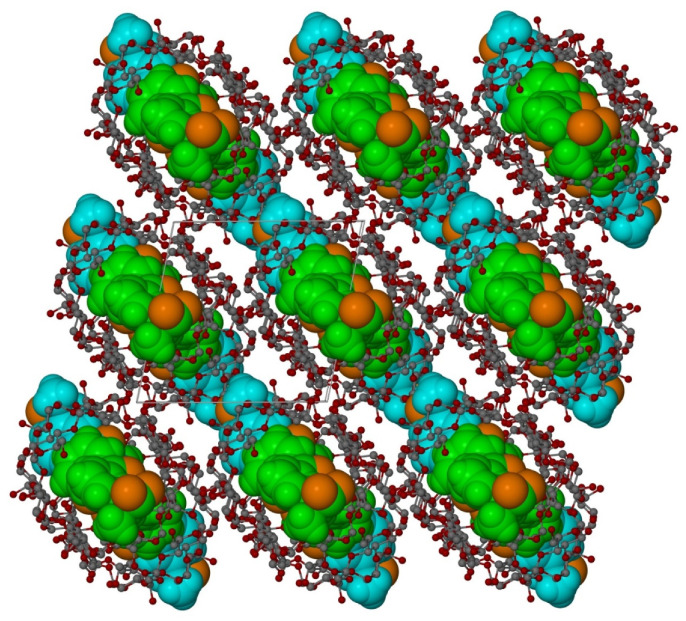
Autostereogram of the crystal structure in b-direction. Only one KP conformation for every blue and green molecule is shown.

In both KP molecules, a disorder is observed with two orientations of each KP molecule: for the molecules A and C, the occupation ratio is 0.658(4):0.342(4); whereas, for B and D, the occupation ratio is 0.387(4)/0.613(4) (Figure 5). One of KP molecules is completely immersed inside one β-CD molecule, whereas another KP molecule is partly outside of another β-CD molecule (Figure 6). As a consequence, the β-CD channels are slightly shifted from linearity (compare Figure 6 and Figure 7 and Figures S2–S4). In Figure 6 and Figure 7, the KP molecules are shown in space filling, whereas the β-CD molecules are shown in balls-and-sticks mode. In these figures, for the sake of clarity, water molecules present in spaces between the β-CD molecules have been omitted.

**Figure 8:**
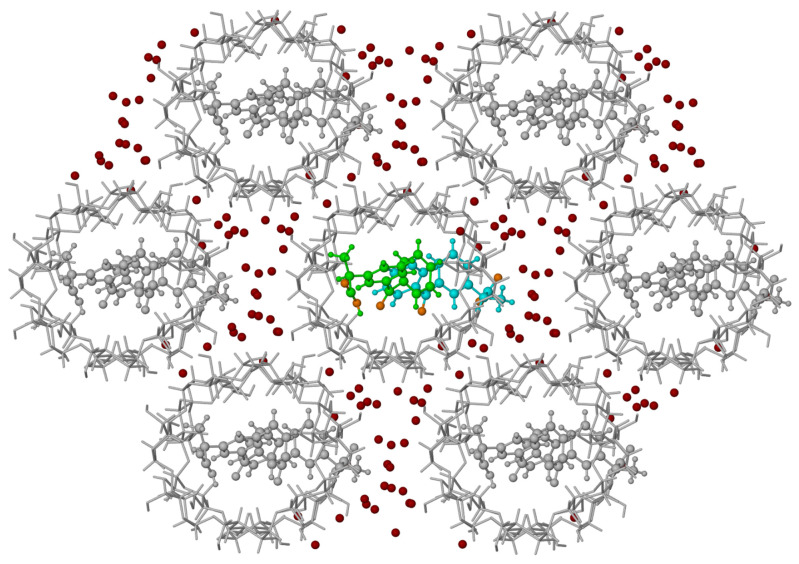
View of crystal packing along the b-direction showing a close neighbourhood of 2:2 KP/β-CD (β-cyclodextrin) inclusion complex. Only O-atoms of water molecules are shown.

Each β-CD channel is surrounded by six additional channels. The direction of the channels is approximately in the *b*-direction of the crystal (Figure 7, Figure 8, and Figure S3). Both host and guest (KP) molecules have both hydrophilic and hydrophobic areas. The expected channel structure, formed by β-CD molecules has a hydrophilic exterior and, therefore, spaces between the neighbouring channels are filled with water molecules bonded to β-CD molecules via hydrogen bonds (Figure 8). There are also hydrogen bonds connecting β-CD molecules directly to each other, without the participation of water molecules. In contrast, the interior of β-CD molecules is hydrophobic and, therefore, the hydrophobic guest parts connect with these areas, whereas the hydrophilic guest areas remain outside. In spaces between channels, there are molecules of water that join channels together by hydrogen interactions linking the hydroxyl group of β-CD molecules of the neighbouring channels. There are 22 molecules of water in the independent part of the unit cell (Figure 8). In Figure 8, the central aggregate (two β-CD molecules filled with two KP) shown in full colour is surrounded by a water layer and six similar aggregates (shown in grey). 

### 3.3. NMR Studies

NMR spectroscopy is an excellent tool that has been used for many years to observe the phenomenon of complexation between CD and guest. The typical effect accompanying CD–guest complexation that can be observed in NMR spectrum expresses itself in the chemical shift changes of the proton signals of the CD and guest, compared to the chemical shift of the corresponding signals of the uncomplexed CD and guest. Analysis of the observed changes provides information on CD–guest interactions. 

#### 3.3.1. One-Dimensional (1D) ^1^H NMR Self-Titration Study of KP in Aqueous Solutions

**Figure 9:**
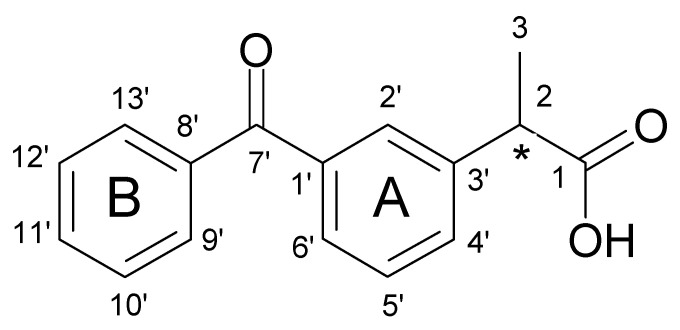
The structure of KP (racemic) with numbering of atoms.

Based on the ^1^H NMR spectrum recorded in the quantitative regime, the concentration of a saturated solution of racemic KP in D_2_O, pH 4.6 was determined to be 0.5 mM. The experimental ^1^H and ^13^C NMR data for a saturated aqueous solution of KP are shown in Table 2. The assignments were based on the analysis of ^1^H-^1^H COSY, ^1^H-^13^C HSQC, and ^1^H-^13^C HMBC spectra. The numbering of the atoms is presented in Figure 9. NMR data for KP dissolved in organic solvents, as well as in D_2_O with the addition of NaOH (pH ≈ 9) [47,48], are available in the literature. However, the differences in assignments of some proton signals obtained in our study, compared to the literature, persuaded the presentation of complete NMR data for racemic KP in D_2_0, pH 4.6.

Rousen and al. [49] proved the dimerisation effect for KP sodium salt in D_2_O solution based on 1D ^1^H NMR self-titration experiments. The concentration was varied between 0.5–5.8 mM. Dimer formation induced shielding effects of the signals of aromatic protons (0.06–0.09 ppm), as well methyl group protons (0.03 ppm).

**Figure 10:**
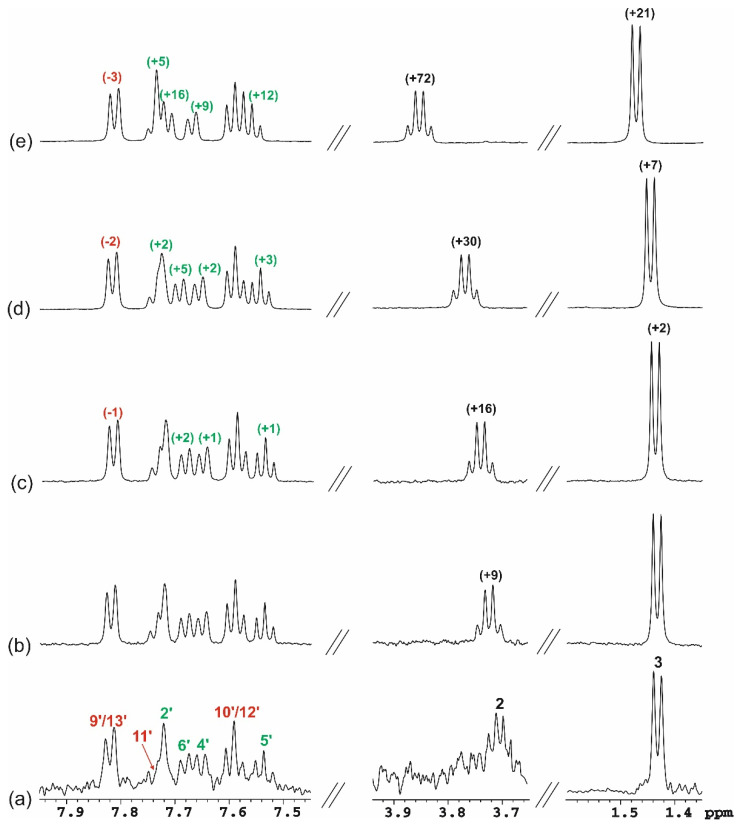
The aromatic (7.40–8.00 ppm) and aliphatic (3.65–3.95 ppm and 1.35–1.60 ppm) fragments of the ^1^H NMR spectra of racemic KP aqueous solutions: C_KP_ = 0.001 mM (**a**), C_KP_ = 0.050 mM (**b**), C_KP_ = 0.100 mM (**c**), C_KP_ = 0.340 mM (**d**), C_KP_ = 0.500 mM (**e**); in brackets. Δδ are given (Δδ = δ_obs_ − δ_0_ is the chemical shift change of proton signals of KP, δ_obs_ = the chemical shift of proton signals of KP at given concentration, δ_0_ = the chemical shift of proton signals of KP at 0.001 mM; positive values indicate high-frequency shifts, [Hz]); chemical shift changes of proton signals of aromatic rings A and B are marked in green and red, respectively; temperature of 25 °C.

To investigate whether the dimerisation process also takes place for racemic KP at concentrations <0.5 mM, 1D ^1^H NMR self-titration experiments were performed for aqueous solutions with concentrations from 1–500 µM. Figure 10 presents the ^1^H NMR spectra of racemic KP in the aqueous solution of different concentrations. Within the tested concentration range with increasing concentration, significant high-frequency chemical shift change was observed for aliphatic proton CH, up to +72 Hz (0.14 ppm). Smaller, but also high frequency shifts were observed for the signal of CH_3_ group protons (+21 Hz) and aromatic protons of ring A (from +5 to +16 Hz). In the whole range of concentrations, significant chemical shifts changes were not observed for the proton signals of ring B. It is worth noting that, in the range of 1–100 µM, the observed changes are actually negligible, which indicates that KP is present as a monomer. Dimer formation takes place in concentrations above 100 µM and this process induced high-frequency chemical shift changes of both aliphatic and ring A proton signals.

#### 3.3.2. KP/β-CD Complexes

The obtained powder samples were dissolved in water and analysed by NMR. Obviously, upon dissolution, the complexes will decompose and an equilibrium between complexed and uncomplexed species of both KP and β-CD will be established according to the binding constant of this system in solution. Although, in this way, we lose the ability to determine many parameters that characterise powder samples, we can easily obtain information about their composition, i.e., determine the molar ratio of KP and β-CD. From this information, one can conclude with a high degree of probability information about the stoichiometry of the complex in the solid state. 

Three mg of the powder obtained by the co-precipitation (sample **1**) or evaporation (sample **2**) methods and 1.2 mg of the powder obtained by the heating-under-reflux (sample **3**) method were completely dissolved in 0.6 mL of D_2_O. The spectra were recorded in the quantitative regime and the concentration, as well as molar ratio of KP and β-CD in the tested solutions, were determined relative to the quantified DSS added (Table 3). Spectral analysis showed that all samples contain 15–20% water. In the samples obtained by methods **1** and **2,** there were large excesses of β-CD in relation to KP, 10 and 75 times, respectively. This may indicate that when methods **1** or **2** were used to obtain KP/β-CD complex, co-precipitation of free β-CD in large amounts occurs, in addition to precipitation of the complex. A sample obtained by method **3** contained equimolar amounts of KP and β-CD. Therefore, it seems that, among the applied methods, only heating-under-reflux is suitable for obtaining the KP/β-CD complex. It can be suggested that the stoichiometry of the obtained complex is 1:1.

**Figure 11:**
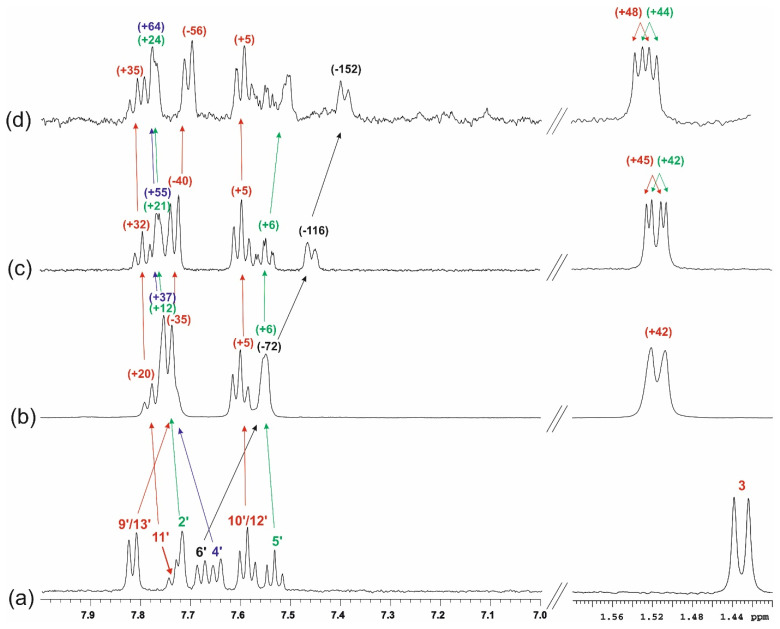
The fragments of the ^1^H NMR spectra (7.0–8.0 ppm and 1.4–1.6 ppm) of: racemic KP aqueous solutions, C_KP_ = 0.050 mM (**a**), sample **3**, C_β-CD_ = C_KP_ = 1.22 mM (**b**), sample **1**, C_β-CD_ = 3.42 mM, C_KP_ = 0.34 mM (**c**), sample **2**, C_β-CD_ = 3.73 mM C_KP_ = 0.05 mM (**d**); in brackets, Δδ are given (Δδ = δ_obs_ − δ_0_ is the chemical shift change of proton signals of KP, δ_obs_ = the chemical shift of proton signals of KP at the presence of β-CD at given concentration, δ_0_ = the chemical shift of proton signals of KP at 0.050 mM; negative values indicate low-frequency shift, (Hz)); chemical shift changes of aromatic protons of rings A and B are marked in green and red, respectively; temperature of 25 °C.

**Figure 12:**
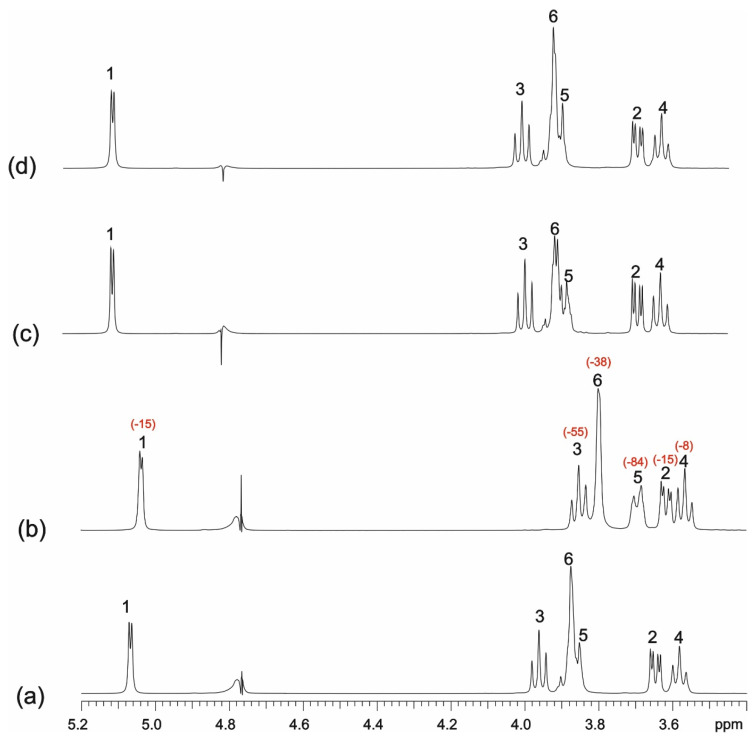
The fragments of the ^1^H NMR spectra (3.4–5.2 ppm) of aqueous solutions of: β-CD, C_β-CD_ = 0.50 mM (**a**), sample **3,** C_β-CD_ = C_KP_ = 1.22 mM (**b**), sample **1**, C_β-CD_ = 3.42 mM, C_KP_ = 0.34 mM (**c**), sample **2**, C_β-CD_ = 3.73 mM, C_KP_ = 0.05 mM (**d**); in brackets, Δδ are given (Δδ = δ_obs_–δ_0_ is the chemical shift change of proton signals of β-CD, δ_obs =_ the chemical shift of proton signals of β-CD at the presence of KP at given concentration, δ_0_ = the chemical shift of proton signals of β-CD at 0.50 mM; negative values indicate low-frequency shift, (Hz); temperature of 25 °C.

The experimental ^1^H and ^13^C NMR data for KP in presence of β-CD (**3**) are displayed in Table 4. Figure 11 and Figure 12 show the ^1^H NMR spectra of aqueous solutions of KP/β-CD complexes obtained by the three methods. Analysis of the ^1^H NMR spectra of samples **1–3** confirmed the formation of complexes between KP and β-CD in aqueous solution. For proton signals of KP in the presence of β-CD, both low- and high-frequency chemical shifts changes were observed (Figure 11). 

For KP, in sample **3** (equimolar solution) (Figure 11b), the largest low-frequency shifts were observed for H6′ proton (−72 Hz). The low-frequency effect was also observed for H9′/13′ protons, but it was much smaller (−35 Hz). For the remaining proton signals of KP, high-frequency effects were observed. The relatively large effect was observed for the H4′, H11′, and CH_3_ group protons (37, 20, and 42 Hz, respectively). For H2′, the observed effect was 12 Hz and for H5′ and H10′/12′, approximately 5 Hz. The observed effects, in terms of the direction of changes, are in agreement with the literature data [47] obtained for an equimolar mixture of KP and β-CD in an aqueous solution of pH 9. However, it should be noted that the low-frequency effects observed in the current study for H6′ and H9′/13′ are definitely smaller than those presented in the literature. This appears to be due to the pH difference of the solutions and not to the different sample preparation methods.

Obviously, when the CD concentration increases, the observed chemical shift changes of the guest proton signals are greater. For example, low-frequency shifts observed for H6′ proton was −116 Hz for sample **1** (Figure 11c), compared to −72 Hz for sample **3** (Figure 11b).

It is worth noting that in the spectra of samples **1** and **2**, in which there a large excess of β-CD in relation to the racemic KP existed, there were separate signals of CH_3_ group protons (at 1.5 ppm) for the (*S*)- and (*R*)- isomers. Direct evidence for the chiral recognition of the applied chiral selector for NMR measurement is parameter ΔΔδ, which denotes the chemical shift differences between the selected signals of (*R*)- and (*S*)-enantiomer complexes. For sample **2** (Figure 11d), in which there was a 75-fold excess of β-CD in relation to KP, the parameter ΔΔδ is approximately 4 Hz. This indicates that the applied measurement conditions were not appropriate for the enantiomeric separation of KP, but that was not the purpose of this work. Moreover, the signal of the CH aliphatic group of KP occurred in a similar range of the ^1^H NMR spectrum as the β-CD proton signals, which resulted in overlapping signals. Therefore, chemical shift changes of this signal were not possible to observe.

When the guest is included into the CD cavity, significant chemical shift changes of the proton signals of β-CD are usually observed. The low-frequency chemical shift changes for the H5 and H3 protons of CD; that is, the signals of the hydrogens located on the inner side of the CD cavity are diagnostic. Considering this, it was easy to see that the chemical shift changes of the signals of β-CD protons of sample **3** confirmed the formation of the inclusion complex KP/β-CD in solution (Figure 12b). The greatest effects were observed for the H5 protons of β-CD (−84 Hz) and a slightly smaller for the other internal H3 protons (−55 Hz). Large effects were also observed for the β-CD H6 protons located outside of the host cavity at the “narrow rim” (−38 Hz). In contrast, chemical shift changes for the H1, H2, and H4 “external” protons are significantly smaller (−8 Hz to −15 Hz).

It can be concluded that inclusion complexes are formed between KP and β-CD, which can be seen from the much stronger effects observed for “inner” β-CD protons H5 and H3 than for the others. Moreover, it may be suggested that the total inclusion of the KP molecule into the β-CD cavity takes place as ΔδH3 < ΔδH5. In the ^1^H NMR spectra of samples **1** and **2** (Figure 12c,d), no changes in the chemical shifts of the β-CD proton signals were observed because there was a large excess of β-CD in relation to KP.

#### 3.3.3. Calculating Binding Constants from the Diffusion Coefficients

The binding constants of KP to β-CD were estimated by using the NMR PFGSE experiment. The PFGSE experiment is a sensitive tool for studying the binding of a molecule of low molecular weight, such as KP (*M*_W_ ≈ 254.3 Da), to a β-CD molecule of much higher molecular weight (*M*_W_ ≈ 1135 Da). The diffusion coefficients and estimated binding constant (*K_a_*) of the studied complexes are displayed in Table 5. The estimated *K_a_* values indicated that KP is a rather strongly bound to β-CD. The average value of the binding constant calculated based on *K_a_* values presented in Table 5 is approximately 2400 M^−1^ and is coherent with previously reported values [47,48]. The *K_a_* values for samples **1** and **2** differ from the average value, which may result from an error in determining the diffusion coefficients for KP in solutions with a large excess of β-CD.

## 4. Conclusions

Among the methods used for the preparation of the KP/β-CD complex, only the heating-under-reflux method is suitable. The complex was confirmed by XRPD and NMR.

The successful single-crystal diffraction experiment performed for the monocrystal obtained as the product of the heating-under-reflux method showed the KP/β-CD complex of 2:2 ratio incorporating an additional 22 molecules of water per complex. Crystallographic data were deposited at Cambridge Structural Data Centre under the number CCDC 2085385. 

Based on NMR studies, it was found that, in the samples obtained by the co-precipitation (**1**) and evaporation (**2**) methods, there were large excesses of β-CD in relation to KP (10 and 75 times, respectively). The sample obtained by the heating-under-reflux method (**3**) contains equimolar amounts of CD and KP. There is a high probability that the stoichiometry of such obtained complexes is 1:1.

Based on NMR studies, it could also be concluded that the inclusion complexes between KP and β-CD are formed in an aqueous solution, as evidenced by chemical shift changes observed for proton signals of KP. The low-frequency chemical shift changes observed for “internal” β-CD protons H5 and H3 also confirm this conclusion. Moreover, it may be suggested that the total inclusion of the KP molecule into the β-CD cavity takes place as ΔδH3 < ΔδH5. 

The estimated binding constants of KP and β-CD were approximately 2400 M^−1^, indicating that KP is quite strongly associated with β-CD.

The association of KP in aqueous solutions was studied using 1D ^1^H NMR self-titration experiments. It was found that the formation of KP dimer already occurs at the concentration of 100 µM.

## Figures and Tables

**Table 1:** Crystal data and structure refinement parameters.

Empirical formula	C_58_H_106_O_49_
Formula weight	1587.42
Temperature	297(2) K
Wavelength	1.54184 Å
Crystal system	Monoclinic
Space group	*P*2**_1_**
Unit cell dimensions	a = 15.1158(3) Å
	b = 32.3308(7) Å
	c = 15.5546(2) Å
	ß = 101.966(2)°.
Volume	7436.4(2) Å^3^
Z	4
Density (calculated)	1.418 mg/cm^3^
Absorption coefficient	1.085 mm^−1^
F(000)	3384
Crystal size	0.177 × 0.222 × 0.451 mm^3^
θ range for data collection	2.733 to 64.506°
Index ranges	−17 ≤ h ≤ 17, −37 ≤ k ≤ 37, −18 ≤ l ≤ 17
Reflections collected	129291
Independent reflections	24,889 [*R_int_* = 0.0376]
Completeness to theta = 64.506°	99.7%
Data/restraints/parameters	24,889 / 2607 / 2212
Goodness-of-fit on F^2^	1.075
Final R indices [I > 2σ(I)]	*R*_1_ = 0.0661, *wR*_2_ = 0.1806
R indices (all data)	*R*_1_ = 0.0708, *wR*_2_ = 0.1889
Absolute structure parameter	0.00(4)
Extinction coefficient	n/a
Largest diff. peak and hole	0.691 and −0.440 e·Å^−3^

**Table 2:** The experimental ^1^H and ^13^C NMR chemical shifts δ (ppm) for racemic ketoprofen (KP) in D_2_O, C = 0.5 mM, pH 4.6, temperature of 25 °C.

Numbering of Atom Position (See Figure 9)	Corresponding Atoms Numbering in Crystal Structure (See Figure S1)	δ_1H_ *	δ_13C_^#^
1 (C = O)	C(3)	-	184.0 (H3, H2)
2 (CH)	C(4)	3.84 (*q,* 1H, *J_HH_* = 7.2)	49.6 (H3, H4′)
3 (CH_3_)	C(5)	1.47 (*d,* 3H, *J_HH_* = 7.2)	20.8 (H2)
1′ (C)	C(10)	-	140.2 (H5′)
2′ (CH)	C(11)	7.74 (overlapped, 1H)	132.3 (H2)
3′ (C)	C(6)	-	145.2 (H3, H2, H5′)
4′ (CH)	C(7)	7.67 (*d,* 1H, *J_HH_* = 7.7)	135.6 (H2, H2′)
5′ (CH)	C(8)	7.56 (*dd*, 1H, *J_HH_* = 7.7, 7.7)	131.9
6′ (CH)	C(9)	7.72 (overlapped, 1H)	131.9 (H4′)
7′ (C = O)	C(12)	-	204.0 (H9′/13′, H6′)
8′ (C)	C(14)	-	139.8 (H10′/12′)
9′/13′ (CH)	C(15)/C(19)	7.81 (*dd,* 2H, *J_HH_* = 8.3, 1.4)	133.3 (H9′/13′, H11′)
10′/12′ (CH)	C(16)/C(18)	7.59 (*dd*, 2H, *J_HH_* = 8.3, 7.6)	131.6 (H10′/12′)
11′ (CH)	C(17)	7.74 (overlapped, 1H)	136.5 (H9′/13′)

* in brackets: multiplicity (*d* doublet, *dd* doublet of doublets, *q* quartet), number of protons and proton–proton coupling constants *J_HH_* (Hz) are presented. ^#^ in brackets: the heteronuclear multiple bond diagnostic correlation between given carbon atom and the showing proton(s) are presented.

**Table 3:** The concentration and molar ratio of KP and β-cyclodextrin (β-CD) in the tested aqueous solutions of powder samples obtained by three methods.

Method Used to Obtain KP/β-CD Complex	Concentration	pH	Concentration [mM]		Molar Ration KP:β-CD
			KP	β-CD	
co-precipitation (**1**)	3.0 mg/0.6 mL	5.8	0.34	3.42	1:10
evaporation (**2**)	3.0 mg/0.6 mL	5.8	0.05	3.73	1:75
heating-under-reflux (**3**)	1.2 mg/0.6 mL	5.8	1.22	1.22	1:1

**Table 4:** The experimental ^1^H and ^13^C NMR chemical shifts δ (ppm) for racemic KP in the presence of β-CD (equimolar solution, sample **3**) in D_2_O, temperature of 25 °C.

Numbering of Atom Position (See Figure 9)	δ_1H_ *	δ_13C_^#^
1 (C = O)	-	183.5 (H3, H2)
2 (CH)	3.83 (overlapped, 1H)	49.6 (H3, H2′)
3 (CH_3_)	1.52 (*d,* 3H, *J_HH_* = 7.3)	21.00 (H2)
1′ (C)	-	139.6 (H5′)
2′ (CH)	7.75 (overlapped, 1H)	131.1 (H2)
3′ (C)	-	145.8 (H3, H2, H5′)
4′ (CH)	7.73 (overlapped, 1H)	135.1 (H2, H2′, H6′)
5′ (CH)	7.55 (overlapped, 1H)	131.4
6′ (CH)	7.55 (overlapped, 1H)	131.4 (H4′, H2′)
7′ (C = O)	-	202.5 (H9′/13′, H4′)
8′ (C)	-	139.5 (H10′/12′)
9′/13′ (CH)	7.75 (overlapped, 2H)	132.3 (H9′/13′, H11′)
10′/12′ (CH)	7.60 (*dd*, 2H, *J_HH_* = 7.6, 7.6)	131.2 (H10′/12′)
11′ (CH)	7.78 (overlapped, 1H)	136.2 (H9′/13′)

* in brackets: multiplicity, number of protons, proton–proton coupling constants *J_HH_* (Hz) are presented. ^#^ in brackets: the heteronuclear multiple bond diagnostic correlation between given carbon atom and the showing proton(s) are presented;. Abbreviations: *d,* doublet; *dd,* doublet of doublets.

**Table 5:** The PGSE data for the binding of KP to β-CD ^1^.

Methods	Concentration (mM)		D_OBS-L_ × 10^−10^m^2^s^−1^	D_OBS-CD_ × 10^−10^ m^2^s^−1^	*MF*_[CD·L]_	*K_a_* (M^−1^)
	C_L_	C_β-CD_				
**1**	0.34	3.42	3.10 ± 0.10	2.70 ± 0.01	0.82 ± 0.05	1 460 ± 100
**2**	0.05	3.73	2.74 ± 0.10	2.56 ± 0.01	0.91 ± 0.05	3 320 ± 200
**3**	1.22	1.22	3.59 ± 0.10	2.56 ± 0.01	0.58 ± 0.05	2 480 ± 100

^1^C**_L_** and C_β-CD_ are the total concentration of KP and β-CD, respectively; D_OBS-L_ is the measured diffusion coefficient for KP in the presence of β-CD; D_OBS-CD_ is the measured diffusion coefficient for β-CD in the presence of KP; *MF*_[CD·L]_ is the calculated molar fraction of KP in complexed form; *K_a_* is a binding constant. Measured diffusion coefficients for uncomplexed species are: β-CD (0.28 mM) = 2.60 ± 0.01 × 10^−10^ m^2^s^−1^; β-CD (6.00 mM) = 2.56 ± 0.01 × 10^−10^ m^2^s^−1^; KP (0.5 mM) = 4.94 ± 0.10 × 10^−10^ m^2^s^−1^; estimated errors for *K_a_* values are presented. The diffusion coefficients calculated by SEGWE [50] for KP (monomer), KP (dimer), and β-CD are equal to 4.83 × 10^−10^ m^2^s^−1^, 3.58 × 10^−10^ m^2^s^−1^, and 2.58 × 10^−10^ m^2^s^−1^, respectively. Based on the comparison of the measured diffusion coefficient of KP at a concentration of 0.5 mM (4.94 × 10^−10^ m^2^s^−1^) with those calculated for monomer and dimer, can be assumed with a high probability that the monomeric form of KP is dominant at a concentration of 0.5 mM. The measured diffusion coefficient of KP (4.94 × 10^−10^ m^2^s^−1^) was used to calculate the binding constants presented in this Table.

## Data Availability

The crystal structure has been deposited at the Cambridge Crystallographic Data Centre (deposition number CCDC 2085385). This data can be obtained free of charge via http://www.ccdc.cam.ac.uk/conts/retrieving.html (accessed on 30 June 2021).

## Supplementary Materials

The following are available online. Figure S1: The numbering scheme of KP A molecule [a2,a3]. Analogous numbering schemes were used for molecules B, C and D, respectively, Figure S2: Independent part of the structure visible from two opposite directions a) and b). Water molecules have been removed to improve readability [a2,a3]. Only KP molecules for conformations A and B are shown (see Figure 5). The hydrogen bonds between CD molecules are shown as the dashed lines. On the bottom figure additional CD molecules and one more KP molecule are shown to view packing in the unit cell, Figure S3: Autostereogram [a4] of the crystal structure shown along the [b] direction [a2,a3]. Only more populated KP molecule orientations A and B are presented (see Figure 5), Figure S4: Autostereogram [a4] of the crystal structure shown along the [a] direction [a2,a3]. Only more populated KP molecule orientations A and B are presented (see Figure 5), Figure S5: Visualization [a2,a3] of oxygen atoms of water molecules in spaces between CD molecules. For clarity, some of molecules of CD (shown as sticks) and KP (shown as ball and sticks) are grey to improve visibility, Figure S6: Numbering scheme of CD molecule. Table S1: Bond lengths [Å] Table S2: Bond angles [°] for KP molecules, Table S3: Torsion angles [°] for KP molecules (see Figure S6). Data in green and violet represent rotations of the ring, Table S4: Short O····O contacts.

## Sample Availability

Samples of the compounds are not available from the authors.
