# Supplementary material for: Comprehensive Characterisation of the Ketoprofen-β-Cyclodextrin Inclusion Complex Using X-ray Techniques and NMR Spectroscopy
Source: Molecules. 2021 Jul 5;26(13):4089. doi: 10.3390/molecules26134089 (PMC8271474; doi:10.3390/molecules26134089)
Supplement: Supplementary file 1 [file molecules-26-04089-s001.zip › molecules-1247089-supplementary.pdf]

Supplementary Materials

Comprehensive Characterisation of the Ketoprofen-β-Cyclodextrin Inclusion Complex Using X-Ray Techniques and NMR Spectroscopy

Katarzyna Betlejewska-Kielak [1], Elżbieta Bednarek [2], Armand Budzianowski [3], Katarzyna Michalska [1,*] and Jan K. Maurin [2,3]

[1] Department of Synthetic Drugs, National Medicines Institute, Chełmska 30/34, 00-725 Warsaw, Poland; k.kielak@nil.gov.pl
[2] Falsified Medicines and Medical Devices Department, National Medicines Institute, Chełmska 30/34, 00-725 Warsaw, Poland; e.bednarek@nil.gov.pl (E.B.); j.maurin@nil.gov.pl (J.K.M.)
[3] National Centre for Nuclear Research, A. Sołtana 7, 05-400 Otwock, Poland; Armand.Budzianowski@ncbj.gov.pl
* Correspondence: k.michalska@nil.gov.pl; Tel.: +48-(22)-841-18-88-(369)

3.2. X-ray single-crystal analysis

Because there are not enough data for refinement of all U_{ij} parameters for so large organic structure at once, we implemented several additional procedures in the refinement process. In general, blocking of some parameters enabled improvement of another part of the structure. Then other parameters were blocked and following were improved etc. Such a recursive process was repeated many times during refinement. At the first stage a rigid structure using AFIX 1 instruction at the beginning of the list of atoms and AFIX 0 at the end of the list of atoms in ShelxL [a1] was applied. For further refinement of the structure, we used AFIX 66 for rings in KP, whereas DFIX and FLAT cards were used for C and O atoms. Additionally, automatic antibumping restraints (BUMP) were generated. Very helpful was the SWAT card which is the only one additional parameter at the refining process, but enabled incorporating a diffuse solvent. This improved our refinement of macromolecular structure for very low quantity of X-ray data in comparison to a large number of independent parameters. In that way, we determined two molecules of CD and two molecules of KP with some splitting of positions of some atoms, which suggested some disorder of carboxyl groups of both molecules.

After recursive improvement of the core structure a challenge to solve the problem of solvent water appeared. The space filled with the solvent is very often not ordered in the real crystal. Crystallographer can apply one of three approaches: use a larger number of isotropic oxygen atoms with partial occupation for water molecules or use a smaller number of oxygen atoms with anisotropic displacement parameters. The third method is to use PLATON SQUEEZE procedure to omit disordered molecules of solvent with volume up 30%. The second approach is very often chemically reasonable. We applied the second technique according to "*SHELXL-93 A Program for the Refinement of Crystal Structures*" and "*User guide to crystal structure refinement with SHELXL*", both by George Sheldrick, to find positions of oxygen atoms with satisfactory distances between hydrogen and oxygen atoms using anti-bumping restraints between water molecules. As the first step, the structure (all atoms' parameters of both CD and both KP molecules) was frozen. The instruction FMAP 2 was used to calculate a difference Fourier map. The instruction PLAN 200 2.3 allow to locate good-looking positions for oxygen atoms. Moreover, software automatically monitored too short distances between peaks. There was also applied the instruction CGLS 10 -1. We applied also a card to prevent generating casual bonds between molecules of water (CONN 0 O01W > LAST).

When positions of water molecules were determined, their atom positions were frozen whereas other molecules were allowed to refine. During this process we located all atoms for partially occupied molecules of KP and instead of partial disorder we got partially occupied molecules (numbers 1-19 see Fig. SM1) A and B respectively. The lower occupied conformations of molecules A and B were denoted with the letters C and D, respectively. There are different occupations of two conformers of molecule A and B. We used three PART cards (0, 1 and 2) to control atoms of each molecule to avoid connections between partially occupied molecules. Additionally, we used MOLE cards to distinguish each water molecule.

Letters F and G at the end of labels were used to denote atoms in two different CD molecules. In the numbering of macrocycle, the every, molecule was divided into 7 mers. for the numbering scheme see Fig. SM6.

Water oxygen atoms have numbers O01W – O25W (some of them with partial occupation).

Because the number of data is not sufficient for unrestrained refinement of all U_{ij} parameters, we used also the ISOR 0.01 O01W > LAST card for anisotropic waters with more or less isotropic behavior. At the last stage of refinement according to Reviewer suggestion we applied partial occupation for some of Oxygen atoms in solvent and new atoms of oxygen were add.

To improve the convergence and prevent excessive shifts in consecutive cycles of refinement we used damping up to 10000 (DAMP 1000 was set in the final stage of refinement) to determine right average positions of water molecules in the structure. The final geometry representation of molecules: bond lengths and angles, torsion angles, and intramolecular short contacts were calculated and are summarized in the cif-file and respective tables below.

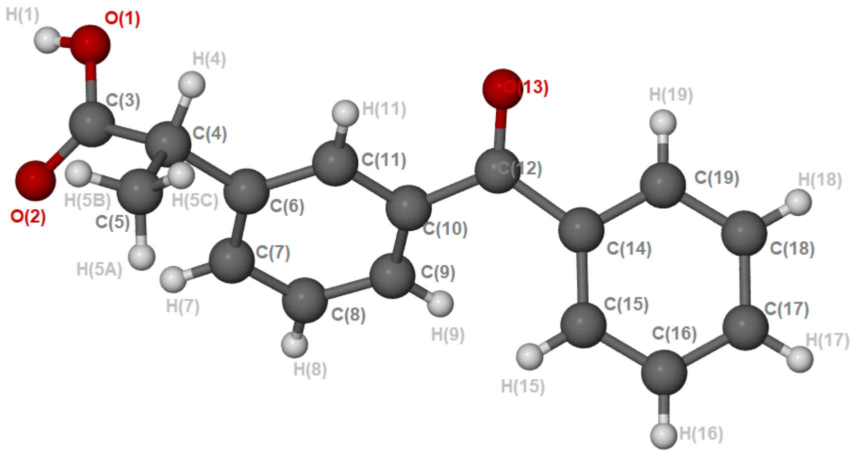

Figure S1. The numbering scheme of KP molecules A, B, C and D, respectively.

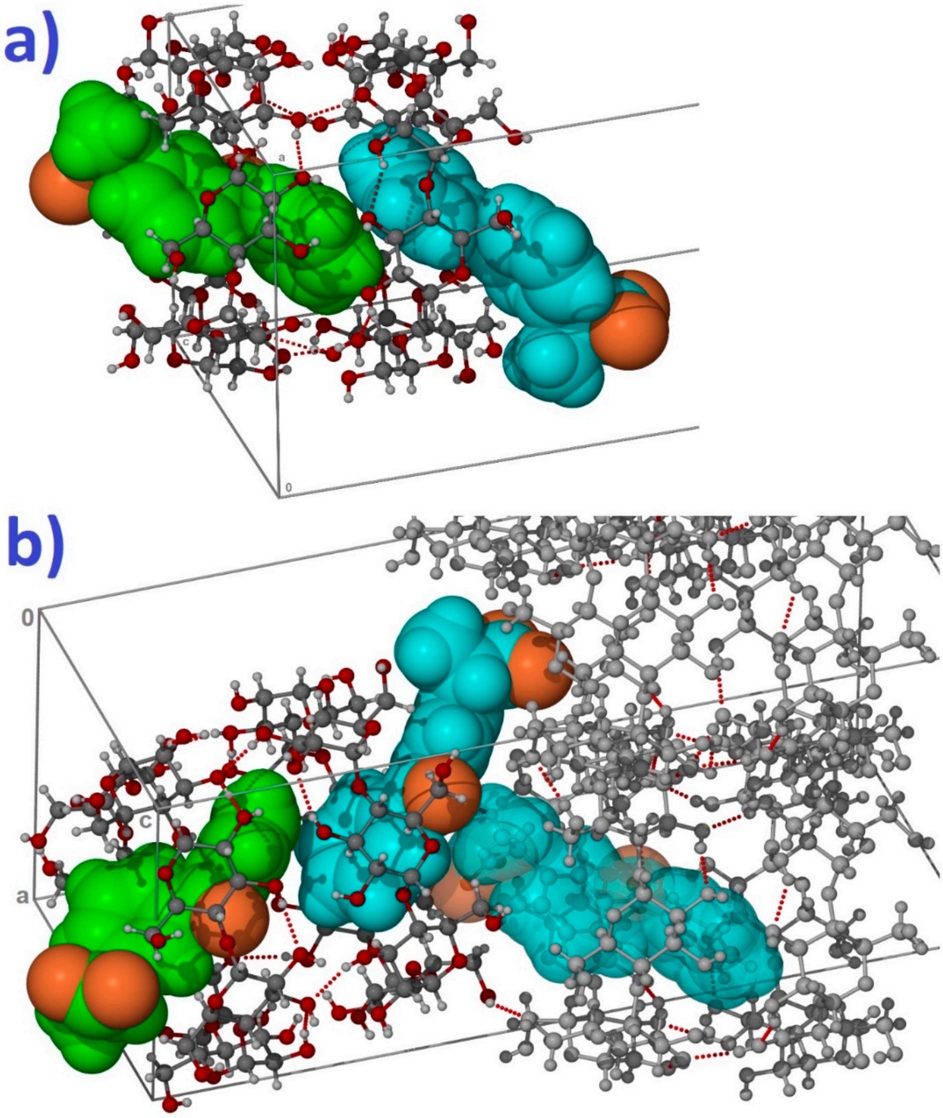

Figure S2. Independent part of the structure visible from two opposite directions (**a**) and (**b**). Water molecules have been removed to improve readability [a2,a3]. Only KP molecules for conformations A and B are shown (see Fig. 5). The hydrogen bonds between CD molecules are shown as the dashed lines. On the bottom figure additional CD molecules and one more KP molecule are shown to view packing in the unit cell.

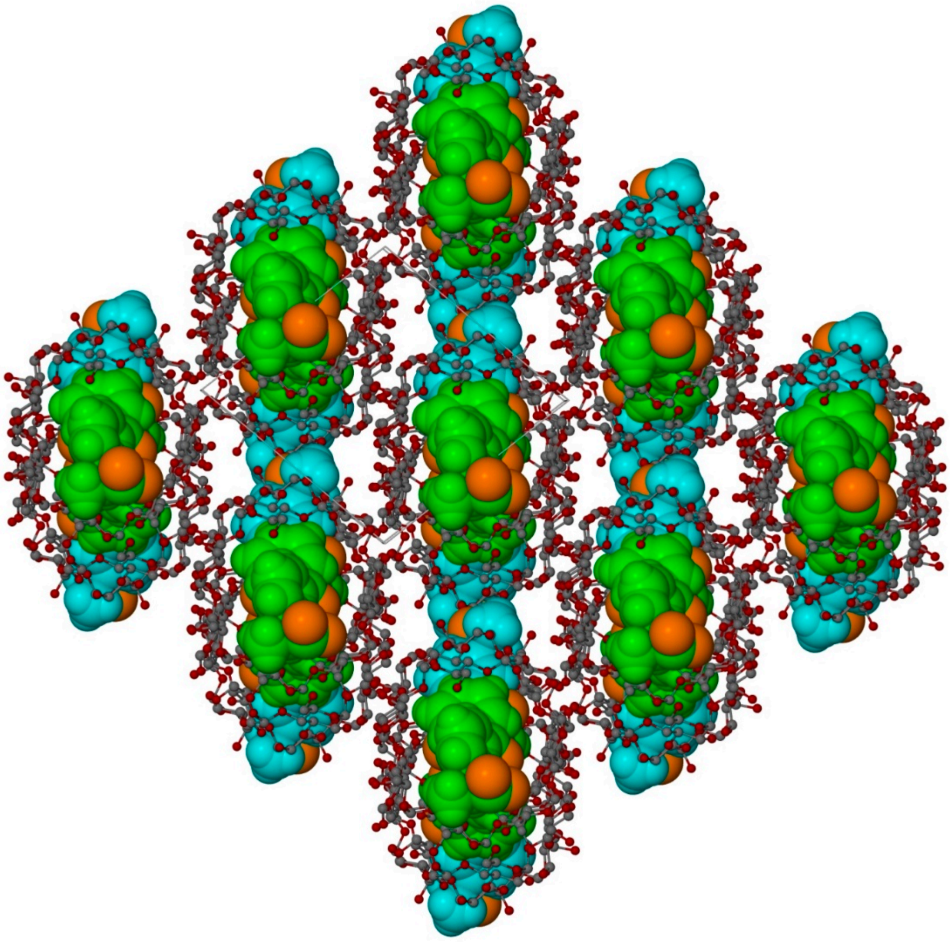

Figure S3. Autostereogram [a4] of the crystal structure shown along the [b] direction [a2,a3]. Only more populated KP molecule orientations A and B are presented (see Fig. 5).

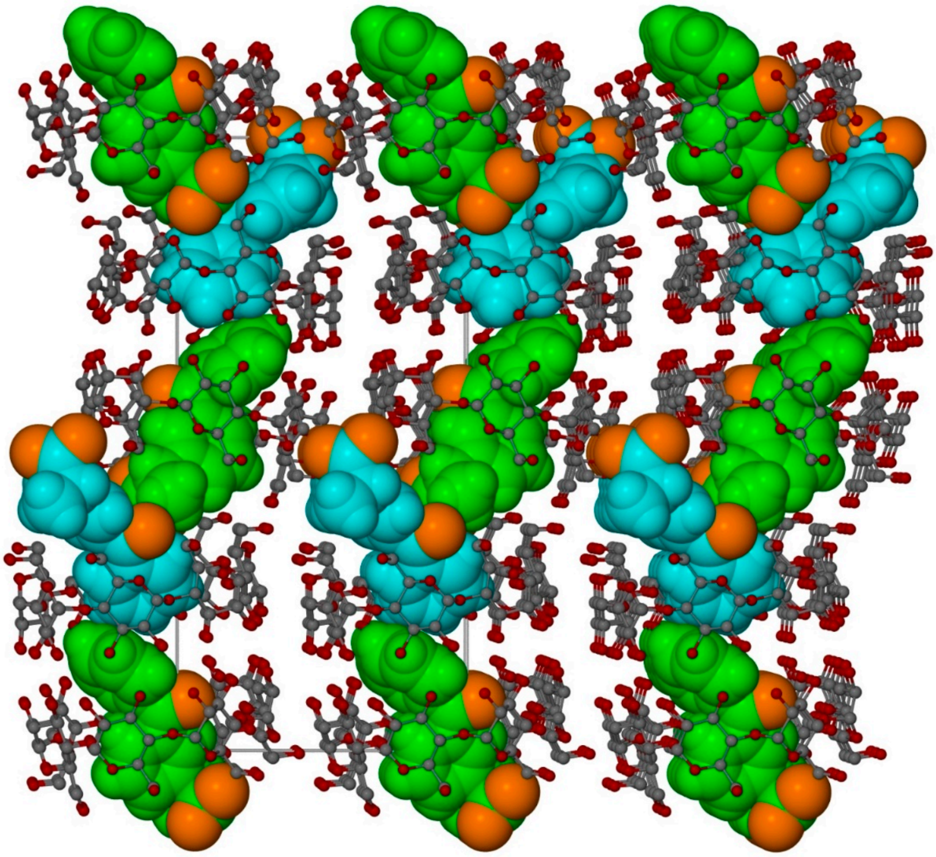

Figure S4. Autostereogram [a4] of the crystal structure shown along the [a] direction [a2,a3]. Only more populated KP molecule orientations A and B are presented (see Fig. 5).

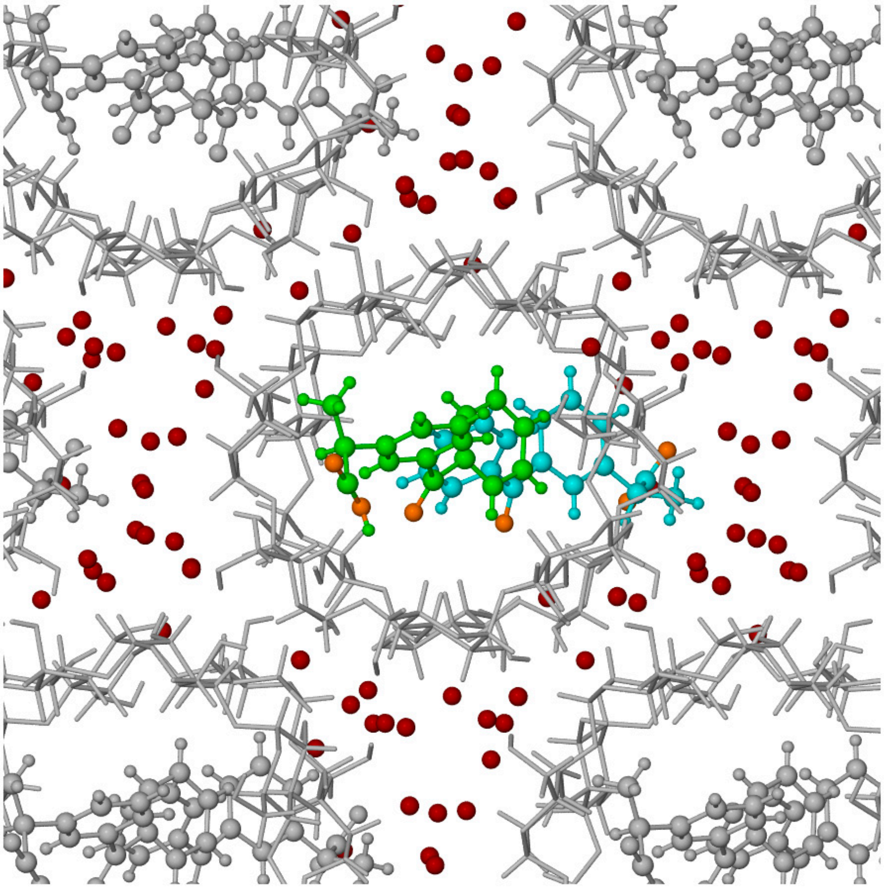

Figure S5. Visualization [a2,a3] of oxygen atoms of water molecules in spaces between CD molecules. For clarity, some of molecules of CD (shown as sticks) and KP (shown as ball and sticks) are grey to improve visibility

Table S1. Bond lengths [Å]

a) for KP molecules (for KP numbering scheme see Fig. SM1).

z=	A	C	B	D
O(1z)-C(3z)	1.244(9)	1.265(13)	1.279(12)	1.295(9)
O(1z)-H(1z)	0.8200	0.8200	0.8200	0.8200
O(2z)-C(3z)	1.236(10)	1.264(13)	1.250(12)	1.241(8)
C(3z)-C(4z)	1.514(9)	1.532(12)	1.510(11)	1.504(10)
C(4z)-C(5z)	1.502(11)	1.540(13)	1.521(12)	1.517(10)
C(4z)-C(6z)	1.509(8)	1.555(11)	1.546(9)	1.529(12)
C(6z)-C(7z)	1.3900	1.3900	1.3900	1.3900
C(6z)-C(11z)	1.3900	1.3900	1.3900	1.3900
C(7z)-C(8z)	1.3900	1.3900	1.3900	1.3900
C(8z)-C(9z)	1.3900	1.3900	1.3900	1.3900

C(9z)-C(10z)	1.3900	1.3900	1.3900	1.3900
C(10z)-C(11z)	1.3900	1.3900	1.3900	1.3900
C(10z)-C(12z)	1.447(8)	1.492(10)	1.484(10)	1.480(11)
C(12z)-O(13z)	1.209(10)	1.232(11)	1.229(12)	1.218(12)
C(12z)-C(14z)	1.477(9)	1.486(9)	1.497(11)	1.496(10)
C(14z)-C(15z)	1.3900	1.3900	1.3900	1.3900
C(14z)-C(19z)	1.3900	1.3900	1.3900	1.3900
C(15z)-C(16z)	1.3900	1.3900	1.3900	1.3900
C(16z)-C(17z)	1.3900	1.3900	1.3900	1.3900
C(17z)-C(18z)	1.3900	1.3900	1.3900	1.3900
C(18z)-C(19z)	1.3900	1.3900	1.3900	1.3900

b) for CD molecule F (for CD numbering scheme see Fig. SM6).

x=	1	2	3	4	5	6	7
O(x1F)-C(x1F)	1.425(4)	1.425(4)	1.428(4)	1.425(4)	1.424(4)	1.434(3)	1.424(4)
C(x1F)-C(x2F)	1.512(5)	1.515(5)	1.510(5)	1.510(5)	1.523(4)	1.507(4)	1.516(5)
C(x1F)-C(x7F)	1.531(5)	1.528(5)	1.538(5)	1.526(5)	1.529(5)	1.528(4)	1.529(5)
C(x2F)-O(x3F)	1.432(4)	1.414(4)	1.416(4)	1.412(4)	1.414(4)	1.430(4)	1.433(4)
C(x2F)-C(x4F)	1.499(5)	1.527(5)	1.518(5)	1.515(5)	1.522(5)	1.518(4)	1.508(5)
C(x4F)-O(x5F)	1.419(4)	1.405(4)	1.421(5)	1.410(5)	1.415(4)	1.427(4)	1.411(5)
C(x4F)-C(x6F)	1.523(5)	1.517(5)	1.530(5)	1.525(5)	1.518(5)	1.523(5)	1.520(6)
C(x6F)-O(x6F)	1.403(4)	1.406(5)	1.402(4)	1.397(4)	1.408(4)	1.417(4)	1.415(5)
C(x6F)-O((*x+1*)1F)	1.413(4)	1.428(5)	1.422(4)	1.424(4)	1.411(4)	1.411(4)	1.419(4)
O(x6F)-C(x7F)	1.431(5)	1.445(5)	1.438(4)	1.440(4)	1.439(4)	1.437(4)	1.429(4)
C(x7F)-C(x8F)	1.512(5)	1.499(6)	1.511(5)	1.501(5)	1.502(5)	1.518(5)	1.510(6)
C(x8F)-O(x9F)	1.409(6)	1.402(9)	1.415(5)	1.420(6)	1.436(6)	1.429(5)	1.381(7)

c) for CD molecule F (for CD numbering scheme see Fig. SM6).

x=	1	2	3	4	5	6	7
O(x1G)-C(x1G)	1.441(4)	1.426(4)	1.436(4)	1.432(4)	1.421(4)	1.432(4)	1.429(4)
C(x1G)-C(x2G)	1.532(4)	1.523(4)	1.516(5)	1.515(5)	1.513(4)	1.528(5)	1.518(5)
C(x1G)-C(x7G)	1.516(5)	1.533(5)	1.530(5)	1.539(5)	1.542(4)	1.525(4)	1.523(5)
C(x2G)-O(x3G)	1.421(4)	1.425(4)	1.409(4)	1.416(4)	1.420(4)	1.414(4)	1.425(4)
C(x2G)-C(x4G)	1.502(5)	1.511(5)	1.519(5)	1.526(5)	1.516(5)	1.521(4)	1.525(5)
C(x4G)-O(x5G)	1.432(4)	1.433(4)	1.408(5)	1.410(4)	1.425(4)	1.418(4)	1.406(5)
C(x4G)-C(x6G)	1.519(5)	1.525(5)	1.514(6)	1.522(5)	1.520(5)	1.520(5)	1.507(6)
C(x6G)-O(x6G)	1.412(4)	1.403(4)	1.405(5)	1.400(4)	1.407(4)	1.392(4)	1.408(5)
C(x6G)-O((*x+1*)1G)	1.413(4)	1.411(4)	1.412(4)	1.419(4)	1.415(4)	1.423(4)	1.414(4)
O(x6G)-C(x7G)	1.441(4)	1.442(5)	1.440(5)	1.434(4)	1.444(4)	1.442(4)	1.443(4)
C(x7G)-C(x8G)	1.510(5)	1.509(6)	1.510(6)	1.504(5)	1.506(5)	1.517(5)	1.504(6)

C(x8G)-O(x9G)	1.435(4)	1.399(8)	1.437(7)	1.423(5)	1.434(5)	1.423(6)	1.423(7)

Table S2. Bond angles [°] for KP molecules.

	A	C	B	D
C(3z)-O(1z)-H(1z)	109.5	109.5	109.5	109.5
O(2z)-C(3z)-O(1z)	124.2(9)	119(2)	173.1(16)	119.5(8)
O(2z)-C(3z)-C(4z)	121.9(9)	128(2)	98.0(13)	130.7(8)
O(1z)-C(3z)-C(4z)	113.8(8)	113.2(14)	88.9(10)	109.1(7)
C(5z)-C(4z)-C(6z)	113.5(8)	116.9(14)	111.4(9)	117.7(8)
C(5z)-C(4z)-C(3z)	116.3(9)	124.2(9)	108.6(6)	94.6(9)
C(6z)-C(4z)-C(3z)	108.9(7)	119.0(14)	115.8(9)	105.0(8)
C(7z)-C(6z)-C(11z)	120	120	120	120
C(7z)-C(6z)-C(4z)	124.2(5)	109.3(7)	116.4(5)	120.8(6)
C(11z)-C(6z)-C(4z)	115.8(5)	129.9(7)	123.5(5)	118.8(6)
C(6z)-C(7z)-C(8z)	120	120	120	120
C(7z)-C(8z)-C(9z)	120	120	120	120
C(10z)-C(9z)-C(8z)	120	120	120	120
C(9z)-C(10z)-C(11z)	120	120	120	120
C(9z)-C(10z)-C(12z)	127.8(5)	118.3(7)	117.2(7)	107.7(8)
C(11z)-C(10z)-C(12z)	112.2(5)	121.7(7)	122.0(6)	132.3(8)
C(10z)-C(11z)-C(6z)	120	120	120	120
O(13z)-C(12z)-C(10z)	125.0(9)	120.2(10)	121.4(13)	131.5(12)
O(13z)-C(12z)-C(14z)	115.0(8)	117.6(10)	125.6(13)	119.5(11)
C(10z)-C(12z)-C(14z)	120.0(6)	122.2(8)	113.0(8)	108.8(8)
C(15z)-C(14z)-C(19z)	120	120	120	120
C(15z)-C(14z)-C(12z)	123.0(5)	119.6(5)	125.8(9)	134.4(8)
C(19z)-C(14z)-C(12z)	117.0(5)	120.4(5)	114.1(9)	105.6(8)
C(14z)-C(15z)-C(16z)	120	120	120	120
C(17z)-C(16z)-C(15z)	120	120	120	120
C(18z)-C(17z)-C(16z)	120	120	120	120
C(17z)-C(18z)-C(19z)	120	120	120	120
C(18z)-C(19z)-C(14z)	120	120	120	120

Table S3. Torsion angles [°] for KP molecules.

z=	A	C	B	D
O(2z)-C(3z)-C(4z)-C(5z)	-50.9(15)	109(2)	6.3(15)	-28.0(12)
O(1z)-C(3z)-C(4z)-C(5z)	132.5(12)	-72(2)	-174.4(11)	161.6(8)
O(2z)-C(3z)-C(4z)-C(6z)	78.9(12)	-72(2)	135.1(13)	81.5(13)
O(1z)-C(3z)-C(4z)-C(6z)	-97.7(11)	107.6(17)	-45.7(11)	-88.9(10)
C(3z)-C(4z)-C(6z)-C(7z)	-43.6(9)	-111.1(12)	-78.9(8)	-51.3(11)
C(5z)-C(4z)-C(6z)-C(7z)	87.7(9)	68.6(17)	47.4(10)	52.2(11)
C(3z)-C(4z)-C(6z)-C(11z)	137.0(6)	79.4(15)	102.2(8)	121.2(8)
C(5z)-C(4z)-C(6z)-C(11z)	-91.7(10)	-101.0(17)	-131.5(9)	-135.3(8)
C(11z)-C(6z)-C(7z)-C(8z)	0	0	0	0
C(4z)-C(6z)-C(7z)-C(8z)	-179.4(5)	-170.7(7)	-178.9(6)	172.5(8)
C(6z)-C(7z)-C(8z)-C(9z)	0	0	0	0
C(7z)-C(8z)-C(9z)-C(10z)	0	0	0	0
C(8z)-C(9z)-C(10z)-C(11z)	0	0	0	0
C(8z)-C(9z)-C(10z)-C(12z)	-179.9(8)	178.8(11)	-169.7(9)	177.9(13)
C(9z)-C(10z)-C(11z)-C(6z)	0	0	0	0
C(12z)-C(10z)-C(11z)-C(6z)	179.9(6)	-178.9(10)	169.2(10)	-178.4(10)
C(7z)-C(6z)-C(11z)-C(10z)	0	0	0	0
C(4z)-C(6z)-C(11z)-C(10z)	179.5(5)	168.6(8)	178.8(6)	-172.6(8)
C(9z)-C(10z)-C(12z)-O(13z)	148.6(9)	161.7(10)	143.1(13)	164.8(15)
C(11z)-C(10z)-C(12z)-O(13z)	-31.4(12)	-19.4(14)	-26.4(18)	-17.2(19)
C(9z)-C(10z)-C(12z)-C(14z)	-30.4(10)	-17.7(13)	-38.0(12)	-10.7(15)
C(11z)-C(10z)-C(12z)-C(14z)	149.6(6)	161.1(7)	152.5(8)	167.4(8)
O(13z)-C(12z)-C(14z)-C(15z)	153.1(8)	140.3(10)	132.8(16)	134.5(15)
C(10z)-C(12z)-C(14z)-C(15z)	-27.8(10)	-40.2(11)	-46.0(13)	-49.4(15)
O(13z)-C(12z)-C(14z)-C(19z)	-28.4(10)	-38.3(13)	-44.4(18)	-47.9(15)
C(10z)-C(12z)-C(14z)-C(19z)	150.7(6)	141.1(9)	136.8(10)	128.2(10)
C(19z)-C(14z)-C(15z)-C(16z)	0	0	0	0
C(12z)-C(14z)-C(15z)-C(16z)	178.4(6)	-178.6(7)	-177.1(10)	177.3(11)
C(14z)-C(15z)-C(16z)-C(17z)	0	0	0	0
C(15z)-C(16z)-C(17z)-C(18z)	0	0	0	0
C(16z)-C(17z)-C(18z)-C(19z)	0	0	0	0
C(17z)-C(18z)-C(19z)-C(14z)	0	0	0	0
C(15z)-C(14z)-C(19z)-C(18z)	0	0	0	0
C(12z)-C(14z)-C(19z)-C(18z)	-178.5(6)	178.6(7)	177.4(9)	-178.0(8)

Table S4. Short distances between Oxygen atoms [Å]: (Symmetry codes: 1: x,y,z; 2: 1-x,1/2+y,2-z; 3: 1-x,-1/2+y,2-z; 4: x,y,-1+z; 5: -1+x,y,z; 6: 1-x,1/2+y,1-z; 7: -x,1/2+y,2-z; 8: -x,-1/2+y,1-z; 9: 1-x,-1/2+y,1-z; 10: -x,-1/2+y,2-z; 11: 1+x,y,1+z; 12: x,y,1+z; 13: -x,1/2+y,1-z; 14: x,y,-1+z; 15: 1+x,y,z; 16: -x,1/2+y,-z+2; 17: -x+1,1/2+y-1,-z+2; 18: -x+1,1/2+y-1,-z+1). The table is divided into short sections a) – g) with descriptions of what the data refer to.

a) Short contacts for KP molecules A, B, C and D:

O1A	1	O76G	2	3.21(1)
O1A	1	O79G	2	2.807(9)
O1A	1	O01W	1	2.62(1)
O1C	1	O01W	1	2.61(2)
O1C	1	O16W	1	3.03(2)
O1B	1	O59F	17	3.26(2)
O1D	1	O59F	17	2.590(6)
O2A	1	O01W	1	3.33(1)
O2A	1	O16W	1	2.92(2)
O2B	1	O13A	17	3.14(2)
O2C	1	O79G	2	2.81(2)
O2C	1	O18W	1	3.35(3)
O2D	1	O59F	17	3.33(8)
O13A	1	O2B	2	3.14 (2)
O13C	1	O2B	2	3.47(2)
O13B	1	O21G	1	3.31(1)

b) Intramolecular short contact in CD molecule F:

O13F	1	O75F	1	2.837(4)
O23F	1	O15F	1	2.780(4)
O33F	1	O25F	1	2.839(4)
O43F	1	O35F	1	2.769(4)
O53F	1	O45F	1	2.824(4)
O63F	1	O55F	1	2.766(4)
O73F	1	O65F	1	2.782(4)

c) Intramolecular short contact in CD molecule G:

O13G	1	O75G	1	2.780(4)
O23G	1	O15G	1	2.756(4)
O33G	1	O25G	1	2.825(4)

O43G	1	O35G	1	2.758(4)
O53G	1	O45G	1	2.838(4)
O63G	1	O55G	1	2.719(4)
O73G	1	O65G	1	2.747(4)

d) Short Intermolecular contacts between two molecules of CD of the unit cell:

O13G	1	O35F	1	3.090(4)
O13G	1	O33F	1	2.783(4)
O23G	1	O25F	1	3.118(4)
O23G	1	O23F	1	2.845(4)
O33G	1	O15F	1	3.020(4)
O33G	1	O13F	1	2.796(4)
O43G	1	O75F	1	3.176(4)
O43G	1	O73F	1	2.944(4)
O53G	1	O65F	1	3.014(3)
O53G	1	O63F	1	2.809(4)
O63G	1	O55F	1	3.197(4)
O63G	1	O53F	1	2.825(4)
O73G	1	O45F	1	3.047(4)
O73G	1	O43F	1	2.841(4)
O15G	1	O33F	1	3.020(4)
O15G	1	O25F	1	2.990(4)
O25G	1	O23F	1	3.123(4)
O25G	1	O15F	1	3.002(4)
O35G	1	O13F	1	3.039(4)
O35G	1	O75F	1	3.044(4)
O45G	1	O73F	1	3.196(4)
O45G	1	O65F	1	3.083(3)
O55G	1	O63F	1	2.997(4)
O55G	1	O55F	1	3.065(4)
O65G	1	O53F	1	3.102(4)
O65G	1	O45F	1	2.949(4)
O75G	1	O43F	1	3.094(4)
O75G	1	O35F	1	3.059(4)

e) Short Intermolecular contacts between -CH2OH groups of CD molecules.

O19F	1	O02W	1	2.830(6)
O19F	1	O18W	1	2.988(1)
O29F	1	O39G	16	3.365(8)
O29F	1	O07W	5	3.35(1)
O29F	1	O19W	1	2.73(1)
O29F	1	O22W	16	2.65(1)
O39F	1	O69F	12	2.778(4)
O39F	1	O14W	1	2.731(6)
O49F	1	O49G	2	2.704(5)
O49F	1	O07W	1	2.78(1)
O59F	1	O1B	2	3.26(2)
O59F	1	O1D	2	2.590(6)
O59F	1	O2D	2	3.329(8)
O59F	1	O2B	2	3.74(2)
O59F	1	O08W	6	2.797(5)
O69F	1	O36F	4	3.264(4)
O69F	1	O39F	4	2.778(4)
O69F	1	O09W	6	2.782(4)
O79F	1	O03W	1	2.85(1)
O79F	1	O14W	4	2.795(6)
O19G	1	O56G	12	3.202(4)
O19G	1	O59G	12	2.803(4)
O19G	1	O16W	17	2.780(9)
O29G	1	O08W	12	2.791(5)
O29G	1	O18W	7	2.75(1)
O39G	1	O29F	7	3.365(8)
O39G	1	O69G	5	3.004(6)
O39G	1	O13W	5	2.838(8)
O39G	1	O19W	7	3.370(1)
O39G	1	O22W	1	2.66(1)
O49G	1	O49F	17	2.704(5)
O49G	1	O09W	1	2.808(5)
O59G	1	O19G	4	2.803(4)
O59G	1	O08W	1	2.735(4)
O69G	1	O36G	15	3.315(5)
O69G	1	O39G	15	3.004(6)
O69G	1	O20W	18	2.74(1)

O79G	1	O1A	17	2.807(9)
O79G	1	O2C	17	2.81(2)
O79G	1	O13W	1	2.836(7)

f) Interactions between molecule CD F and water:

O13F	1	O21W	5	2.872(8)
O23F	1	O11W	1	2.88(1)
O23F	1	O23W	1	3.03(4)
O43F	1	O06W	1	3.10(1)
O43F	1	O23W	15	3.28(4)
O63F	1	O15W	4	3.164(7)
O73F	1	O17W	1	2.988(8)
O73F	1	O24W	1	2.99(2)
O75F	1	O04W	1	2.90(1)
O75F	1	O05W	1	3.296(1)
O75F	1	O21W	5	3.018(9)
O25F	1	O24W	12	2.884(2)
O35F	1	O12W	1	2.829(8)
O45F	1	O23W	15	2.52(4)
O55F	1	O12W	4	3.265(8)
O55F	1	O15W	4	3.285(6)
O55F	1	O21W	1	2.592(8)

g) Interactions between molecule CD G and water:

O13G	1	O15W	1	2.922(6)
O23G	1	O17W	12	2.796(8)
O23G	1	O24W	12	3.38(2)
O43G	1	O01W	8	2.914(9)
O63G	1	O21W	1	3.19(1)
O73G	1	O11W	15	2.831(9)
O73G	1	O23W	15	3.21(3)
O75G	1	O04W	11	2.99(1)
O75G	1	O06W	1	3.13(1)
O75G	1	O15W	1	3.285(6)
O25G	1	O11W	1	2.857(1)
O35G	1	O05W	1	2.49(1)
O45G	1	O17W	1	2.743(8)
O55G	1	O05W	15	3.229(1)
O55G	1	O15W	4	2.695(6)
O55G	1	O21W	1	3.322(9)

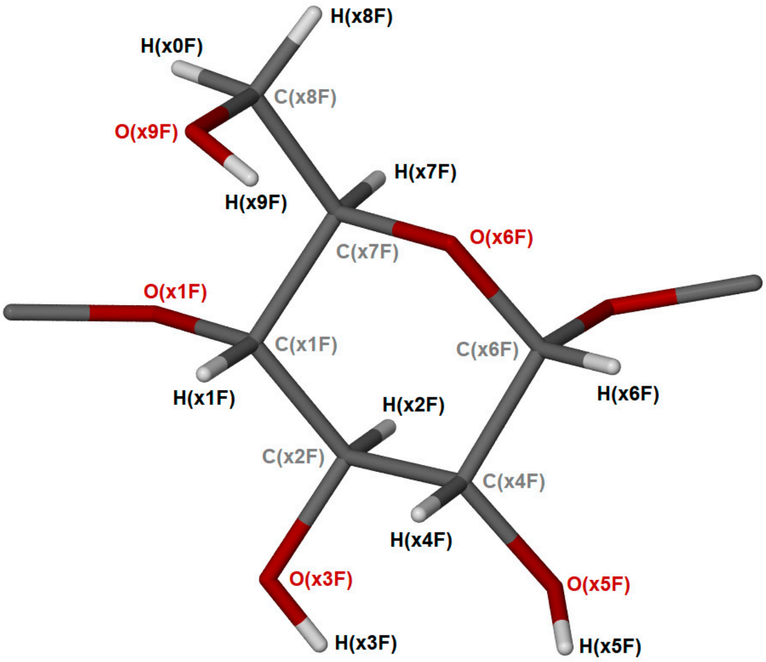

Figure S6. Numbering scheme of CD molecule F. The "x" can be between 1 and 7 in each F and G CD molecule.

References

1 G.M. Sheldrick, Crystal structure refinement with SHELXL, *Acta Cryst.*, **2015**, *C71,* 3–8. http://dx.doi.org/10.1107/S2053229614024218.
2 L.J. Barbour., X-Seed – A Software Tool for Supramolecular Crystallography, *J. Supramol. Chem.* **2001**, *1*, 189–191. http://dx.doi.org/10.1016/S1472-7862(02)00030-8.
3 Persistence of Vision Pty. Ltd. **2004**. Persistence of Vision (TM) Raytracer. Persistence of Vision Pty. Ltd., Williamstown, Victoria, Australia. http://www.povray.org/.
4 A. Katrusiak., Crystallographic autostereograms, *J. Mol. Graph. and Model*. **2001**, *19*, 363–367. https://doi.org/10.1016/S1093-3263(00)00085-1.
